# The role of fungi in heterogeneous sediment microbial networks

**DOI:** 10.1038/s41598-019-43980-3

**Published:** 2019-05-17

**Authors:** Jenny Marie Booth, Marco Fusi, Ramona Marasco, Grégoire Michoud, Stilianos Fodelianakis, Giuseppe Merlino, Daniele Daffonchio

**Affiliations:** 1 King Abdullah University of Science and Technology, Red Sea Research Center, Thuwal, 23955-6900 Saudi Arabia; 2 School of Applied Sciences, Edinburgh Napier University, Edinburgh, UK

**Keywords:** Fungal ecology, Microbial ecology

## Abstract

While prokaryote community diversity and function have been extensively studied in soils and sediments, the functional role of fungi, despite their huge diversity, is widely unexplored. Several studies have, nonetheless, revealed the importance of fungi in provisioning services to prokaryote communities. Here, we hypothesise that the fungal community plays a key role in coordinating entire microbial communities by controlling the structure of functional networks in sediment. We selected a sediment environment with high niche diversity due to prevalent macrofaunal bioturbation, namely intertidal mangrove sediment, and explored the assembly of bacteria, archaea and fungi in different sediment niches, which we characterised by biogeochemical analysis, around the burrow of a herbivorous crab. We detected a high level of heterogeneity in sediment biogeochemical conditions, and diverse niches harboured distinct communities of bacteria, fungi and archaea. Saprotrophic fungi were a pivotal component of microbial networks throughout and we invariably found fungi to act as keystone species in all the examined niches and possibly acting synergistically with other environmental variables to determine the overall microbial community structure. In consideration of the importance of microbial-based nutrient cycling on overall sediment ecosystem functioning, we underline that the fungal microbiome and its role in the functional interactome cannot be overlooked.

## Introduction

The diversity and functions of prokaryote communities in soils and sediments, with particular focus on the specific roles of different groups in element cycling, have been extensively studied^1,2^, spanning nitrogen cycling in deep-sea benthic sediments^3^ and Antarctic Dry Valleys^4^ to phosphorus cycling in hypoxic coastal sediments^5^. Conversely, despite their huge diversity (estimated at several million species), the role of fungi in the overall functional structure of sediment communities is poorly defined, partly due to the limited number of fungal genomes available compared to those of prokaryotes (currently more than 23 000 prokaryotic non-redundant genomes are described compared to just over 1500 for fungi^6,7^). Consequently, the role of fungi is limited to their functional classification as pathotrophs, saprotrophs and symbiotrophs^8,9^.

Having monopolised the lignocellulose decomposition niche, fungi have a central role in soil and sediment ecosystem functioning^10,11^. Yet, their interactions with bacteria in substrates rich in lignocellulose are not necessarily competitive and mechanistic studies have revealed the importance of fungi in providing several central services to prokaryotic communities^11^. Those most widely known include fungal highways, whereby bacteria are able to move along fungal hyphae (a spatial service^12,13^), lignocellulose degradation (a metabolic service^14,15^) and the provision of water and nutrients (a nutritional service^16^). In highly heterogeneous sediments, such as those in intertidal regions, the active ecological role of fungi has been overlooked and considering that sediment microbial communities are connected by synergistic, antagonistic or neutral relationships^17,18^ the interaction of the three main components (fungi, bacteria and archaea) are key to understanding sediment processes and ecosystem functioning.

Recently, communities of fungi and bacteria in woodland and pasture soils were reported to form distinct associations unrelated to local environmental conditions^19^. That study highlights the concept that components of soil and sediment microbial communities should not be oversimplified and, rather, considered together in order to understand true microbial structure and function. We reason that the role of fungi in the functional services of a sediment microbial community should be better defined and we raise the hypothesis that the fungal microbiome plays a role in the overall coordination of whole microbial communities, controlling the structure of sediment microbial functional networks. To test our hypothesis, we considered a sediment environment with high niche heterogeneity, specifically intertidal sediment within the mangrove ecosystem, subject to high levels of bioturbation by burrowing animals. These sediments harbour (1) highly anaerobic niches, due to the cyclical submersion by tidal waters^20^; (2) aerobic niches at the sediment water/air interface; and (3) organic carbon-rich niches where bioturbating animals passively and/or actively store organic carbon in the form of litter harvested from the mangrove floor^21^. We explored the assembly of the fungal, bacterial and archaeal communities, across the geochemical gradients occurring around the burrows excavated by the herbivorous crab *Neosarmatium africanum*, that removes, through consumption or burial, up to 80% of annual litter fall^22^.

## Results

### Burrow effects on bacterial, archaeal and fungal OTU assemblages

**Figure 1:**
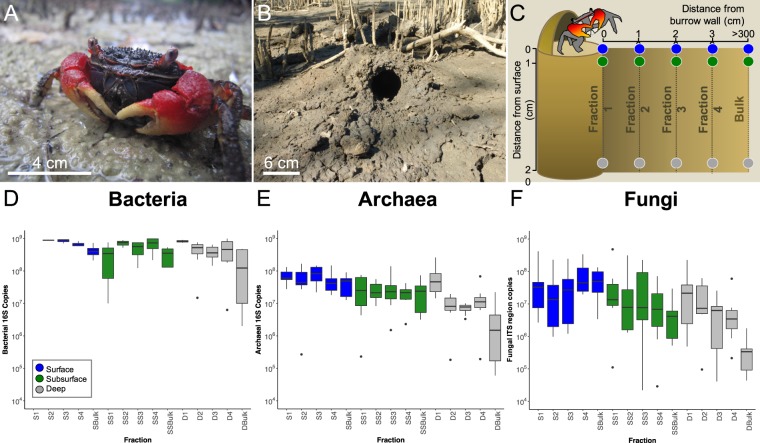
(**a**) The sesarmid crab *Neosarmatium africanum* and (**b**) the typical burrow structure with a hood at the burrow entrance. (**c**) Sampling design adopted to dissect the microbial structure at three depths and at five distances away from the burrow wall. (**d–f** ) qPCR for the total bacterial, archaeal and fungal communities. All 16S rRNA and ITS1 copies are normalized per g of sediment.

We sampled eight burrows, comprising five sediment fractions (1, 2, 3, 4 and bulk) along a horizontal gradient away from the burrow, at three depths (surface, subsurface, deep; total of 15 fractions per burrow; see Fig. 1a–c and Methods for further detail).

Quantitative PCR (qPCR) analysis revealed a significant effect of ‘Depth’ (ANOVA, F_2,104_ = 15.97, *P* < 0.001) and ‘Fraction’ (ANOVA, F_4,104_ = 8.84, *P* < 0.001; Fig. 1d) on the number of copies of the bacterial 16S rRNA gene per gram of sediment, being highest in the surface and at the burrow wall at each ‘Depth’. We detected a significant ‘Depth × Fraction’ interaction on the number of copies of the archaeal 16S rRNA gene (ANOVA, F_8,105_ = 2.37, *P* < 0.05) and fungal ITS region (ANOVA, F_8,104_ = 2.11, *P* < 0.05) per gram of sediment, both decreasing away from the burrow wall at each ‘Depth’ (Fig. 1e,f; Supplementary File 1).

**Figure 2:**
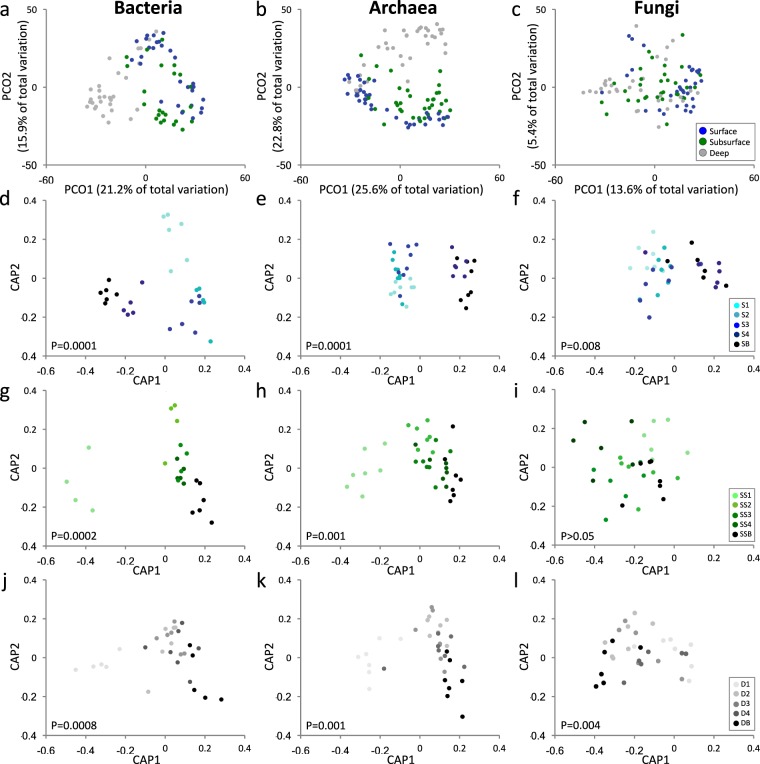
Principal coordinates analysis (PCoA) of bacterial, archaeal and fungal communities in surface, subsurface and deep sediment (**a–c**). Constrained analysis of the canonical axes of the principal coordinates (CAP) showing the differences between each ‘Fraction’ at each ‘Depth’ for bacteria, archaea and fungi. CAP statistic is reported at the bottom of each graph (**d–l**).

Analysis of microbial community composition revealed a significant interaction of ‘Depth × Fraction’ on bacterial, archaeal and fungal OTU assembly (GLM, *P* < 0.01 in all cases, Supplementary Table S1). Bacterial and archaeal communities inhabiting surface, subsurface and deep sediment were well segregated, explaining up to 37.1% and 48.4%, respectively, of the total variability; while fungal communities were less segregated with 19% of total variability explained (PCoA, Fig. 2a–c). Further analysis revealed a significant effect of ‘Fraction’ on bacterial, archaeal and fungal OTU assemblages in surface, subsurface and deep sediment (CAP, *P* < 0.01 in all cases; Fig. 2d–l, Supplementary Table S2), with the exception of fungal communities in the subsurface (CAP, *P* > 0.05).

Bacterial alpha diversity and richness were consistently higher at each ‘Depth’ and in each ‘Fraction’ compared to those of archaea and fungi (Supplementary Fig. S1). A significant interaction of ‘Depth × Fraction’ was observed on archaeal and fungal species diversity and richness only (PERMANOVA, *P* = 0.001 and *P* = 0.01, respectively, for both diversity and richness; Supplementary Fig. S1, Supplementary Tables S3–5, see Supplementary File 1 for further detail). Differences in diversity and richness were reflected in differential community composition at each ‘Depth’ and ‘Fraction’ for bacteria, archaea and fungi (Supplementary Fig. S2, Supplementary Tables S6 and 7, Supplementary File 1).

### Identifying discriminately abundant taxa throughout the burrow wall

Linear discriminant analysis effect size (LEfSe) was used to identify taxa that were significantly more abundant at each depth and distance from the burrow wall (‘Fraction’).

#### Bacteria

Communities of each ‘Fraction’ at each ‘Depth’ had a portion of the community that was significantly more abundant than in other fractions (LEfSe, Wilcoxon *P* value: 0.05, LDA score > 2; Supplementary File 2). Notably, in the surface a significantly higher number of photosynthetic bacteria, such as *Cyanobacteria* (e.g. Class *Oscillatoriales*) and purple non-sulphur bacteria (e.g. sulphur-oxidising families *Rhodospirillaceae* and *Rhodobacteraceae*), were detected in Fraction 4, furthest from the burrow wall, and bulk than in other fractions. In Fraction 1 of the subsurface, many taxa involved in sulphur cycling, such as sulphate reducers (e.g. *Desulfarculus*, *Desulfobulbus* and *Desulfobacteria*) and sulphur reducers (e.g. *Desulfomonil* and *Desulfuromonas*), were significantly more numerous compared to other fractions. Diverse sulphur cycling taxa were also discriminately more abundant at the burrow wall, in Fraction 1, of the deep compared to other fractions at the same depth: sulphate reducers (e.g. *Desulfocapsa*, *Desulfobacter*, *Desulfobulbus* and *Desulfovibrio*), sulphur reducers (*e*.*g*. *Sulfurospirillum* and *Desulfuromonas*) and a sulphur oxidizer (*Sulfurimonas*).

#### Archaea

In surface and subsurface sediment, there was a discriminate abundance of different taxa at each distance from the burrow and bulk sediments (LEfSe; LDA score > 2, Supplementary File 2). In the surface, in Fractions 1 and 2, several methanogenic taxa such as *Methanosarcinaceae* and *Methanobacteriales* were significantly more abundant. The ammonia oxidising *Candidatus Nitrososphaera* (family *Nitrososphaeraceae*) was more abundant in the bulk of both the surface and subsurface. In the subsurface, in Fraction 1, methylotrophic (e.g *Methanomassiliicoccaceae*) and methanogenic taxa (family *Methanosarcinaceae*) were significantly enriched.

#### Fungi

In surface sediment, several taxa were found to be more abundant in bulk sediment (LEfSe; LDA score > 2, Supplementary File 2), specifically taxa belonging to the orders *Diaporthales*, *Agaricomycetes* and *Sordariomycetes*. In the deep, several taxa were discriminately more abundant at the burrow wall in Fraction 1: *Botryosphaeriales* and *Capnodiales* (order *Dothideomycetes*), *Phialophora* (order *Eurotiomycetes*), and the two genera *Cryptococcus* and *Dioszegia* (order *Tremellocyetes*).

Ternary plots showed differential sharing of OTUs among the surface, subsurface and deep in bulk and burrow sediment, with a clearer differentiation in bacteria and archaea (Supplementary Fig. S3). In the burrow sediment, more bacterial, archaeal and fungal OTUs were shared between all depths compared to bulk sediment (Supplementary Fig. S3); for bacteria, there were only a small number of OTUs shared between the surface and deep of bulk sediment, while for archaea we observed a larger number of OTUs to be unique to the deep in the bulk sediment.

The Sloan model showed a significant homogenizing effect, i.e., mixing of sediment in the direction of the subsurface to the surface was observed at the burrow wall (Fraction 1), for bacteria and archaea (Sloan model, R^2^ = 0.41, n = 2715, *P* < 0.001 and R^2^ = 0.54, n = 843, *P* < 0.001, respectively). The fit of the model was negative for fungi and in all other directions concerning bacteria and archaea, i.e., downward within the burrow and at all distances from the burrow, upward at all distances from the burrow and horizontally with increasing distance from the burrow (−0.981 < R^2^ < −0.456).

### Microbial network topology

**Figure 3:**
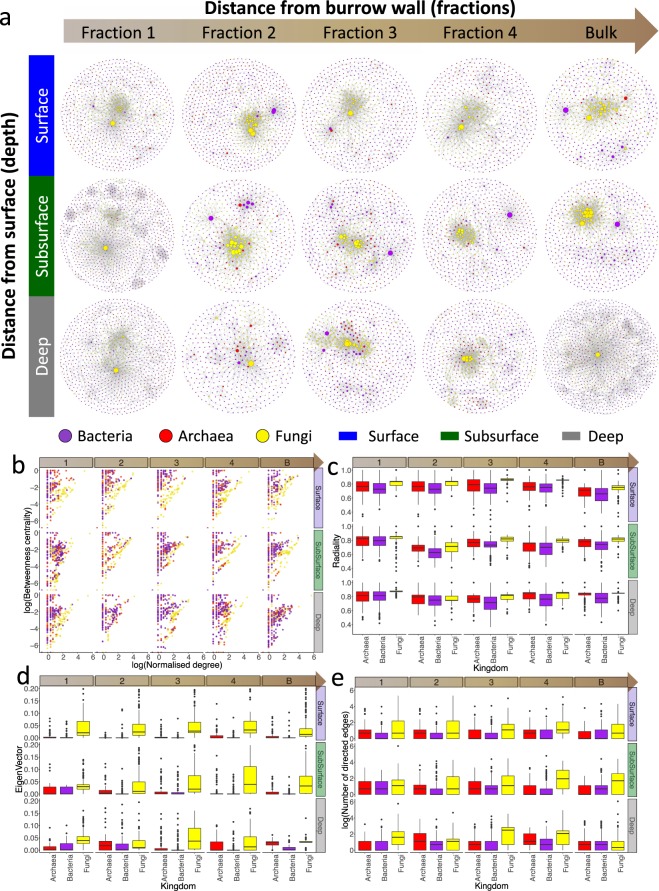
Interkingdom co-occurrence network analysis for each ‘Fraction’-‘Depth’ interaction (**a**). Keystone species analysis: Betweenness centrality vs. node degree of all species in the cross-domain bacterial-archaeal-fungal networks (**b**). Radiality analysis (**c**). Eigenvector analysis (**d**). The number of directed edges (**e**).

Network topological parameters were significantly different for each ‘Fraction’ (distance from the burrow wall) at each ‘Depth’ (Fig. 3; for detail see Fig. S4 in the Supplementary File 1).

Fungi nodes were the dominant hubs across all fractions (Fig. 3) and network centrality measures differed for each ‘Kingdom’ across each ‘Fraction’ at each ‘Depth’. In surface sediment, a significant effect of ‘Kingdom’ was observed on the degree of connection (ANOVA, F_2,3286_ = 110.69, *P* < 0.001; Supplementary Fig. S5), with a higher connectivity of fungi compared to archaea and bacteria. While in the subsurface and deep, where fungi showed a higher degree of connection in each ‘Fraction’ at each ‘Depth’ (except bulk deep) compared to bacteria and archaea, a significant interaction of ‘Fraction × Kingdom’ was observed (respectively, ANOVA: F_2,3373_ = 3.847, *P* < 0.001; F_4,3612_ = 4.307, *P* = < 0.001). A significant interaction of ‘Fraction × Kingdom’ was observed on average shortest path length in surface, subsurface and deep sediment for bacteria, archaea and fungi (respectively, ANOVA: F_8,3286_ = 71.51, *P* < 0.001; F_8,3373_ = 2.497, *P* < 0.05; F_8,3612_ = 6.998, *P* < 0.001; Supplementary Fig. S5). Average path length increased towards bulk sediment in the surface and subsurface, with a higher path length attributed to archaea and bacteria. Closeness centrality in surface sediment decreased towards the bulk for bacteria (‘Fraction × Kingdom’ surface χ2, deviance _8,3286_ = 770.04, *P* < 0.001; Supplementary Fig. S5). In the deep, fungi had higher closeness centrality in Fraction 1 than bacteria and archaea (χ2 deviance _8,3612_ = 720.38, *P* < 0.001). Betweenness centrality was persistently higher for fungi at all depths across all fractions (‘Fraction × Kingdom’: χ2 deviance _8,3286_ = 992.91, *P* < 0.001). In subsurface sediment, a significant effect of ‘Fraction’ (χ2 deviance _4,3383_ = 790.85, *P* < 0.01) and ‘Kingdom’ (χ2 deviance _2,3381_ = 782.87, *P* < 0.01) was observed (Supplementary Fig. S5). Similarly, in the deep a significant effect of both ‘Fraction’ (χ2 deviance _4, 3622_ = 78.20, *P* < 0.001) and ‘Kingdom’ (χ2 _2,3620_ = 773.61, *P* < 0.05) was observed. Edge betweenness measures for each ‘Fraction’ are shown in Fig. S6 (see Supplementary File 1 for further detail).

Keystone nodes analysis (Fig. 3c–e) revealed that fungi in each ‘Fraction’ and ‘Depth’ showed a significantly higher level of radiality (χ2 deviance _16,10245_ = 154.75, *P* < 0.01), eigenvector (χ2 deviance _16, 10153_ = 258.06, *P* < 0.01) and number of directed edges (χ2 deviance _16,10245_ = 793459, *P* < 0.01). This was confirmed by the significance of the relationship between betweenness centrality and degree of connection (Fig. 3b), where fungi consistently had the highest level of degree of connection with the highest level of betweenness centrality compared to bacteria and archaea (GAM; F_8,10316_ = 132.2; *P* < 0.01). Fungal OTUs had the highest degree of connectivity and formed the majority of the keystone nodes in surface, subsurface and deep sediment (having an overall highest degree of connectivity, betweenness centrality and closeness centrality; Table 1). In the surface, in Fraction 1, the largest keystone node was the saprotrophic fungal family *Trichocomaceae* followed by the bacterial keystone node *Pelobacteraceae*. Fungi also formed the majority of keystone nodes in Fractions 2 to 4 (*Sordariomycetes* being a consistently highly connected node). The green non-sulphur bacteria *Thermomicrobia* and the anaerobic sulphate-reducing bacteria *Desulfobulbaceae* formed important nodes in Fractions 2 and 4, respectively (Table 1). In the subsurface (Table 1), fungi such as *Pluteus* (a wood-rotting saprobe) formed significant keystone nodes throughout the burrow wall. Large bacterial nodes included the purple sulphur bacteria *Chromatiales* (the second most important node in Fraction 2) and the ammonia oxidizing bacteria *Candidatus Nitrososphaera*. In the deep (Table 1), fungi formed the majority of the most important keystone nodes. In Fraction 1 these included OTUs belonging to the orders *Sordariomycetes* and *Dothideomycetes*. The bacteria *Piscirickettsiaceae* was also a significant node in Fraction 1 at the burrow wall. In Fraction 2 and bulk sediment, several keystone nodes were formed by members of the archaeal order *Crenarchaeota*. A bacterial OTU belonging to the family *Desulfobacteraceae* was a highly connected central node in Fraction 3. In Fraction 4, a member of the strictly aerobic bacteria family *Hyphomonadaceae* formed an important node.

**Table 1:** Keystones nodes obtained from the network analysis for surface (A), subsurface (B) and deep (C) sediment across each ‘Fraction’ considering the highest level of node degree distribution, closeness centrality and betweenness centrality.

Fraction	Kingdom	Lowest taxa	Betweenness Centrality	Closeness Centrality	Degree
S1	Fungi	*Trichocomaceae*	0.863	0.427	207
	Fungi	Unidentified	0.030	0.278	64
	Bacteria	*Pelobacteraceae*	0.084	0.257	53
	Fungi	*Lyophyllum*	0.005	0.268	49
	Fungi	Unidentified	0.069	0.332	46
	Fungi	Unidentified	0.005	0.267	43
	Fungi	*Ascomycota*	0.004	0.267	42
	Archaea	*Crenarchaeota*	0.125	0.259	41
	Archaea	YLA114	0.106	0.259	37
	Fungi	*Onygenaceae*	0.007	0.322	35
S2	Fungi	Unidentified	0.472	0.272	90
	Fungi	*Sordariomycetes*	0.308	0.260	88
	Bacteria	*Thermomicrobia*	0.115	0.229	71
	Bacteria	*Marinobacter*	0.110	0.229	70
	Fungi	*Ascomycota*	0.075	0.245	58
	Fungi	Unidentified	0.053	0.238	45
	Fungi	Unidentified	0.042	0.237	39
	Fungi	*Aureobasidium*	0.025	0.236	36
	Fungi	*Ascomycota*	0.015	0.236	36
	Fungi	*Onygenaceae*	0.012	0.231	32
S3	Fungi	*Sordariomycetes*	0.741	0.481	149
	Bacteria	*Anaerolineae*	0.120	0.321	59
	Archaea	*Parvarchae*	0.095	0.318	55
	Fungi	*Botryosphaeriaceae*	0.203	0.401	46
	Fungi	*Sordariomycetes*	0.039	0.382	44
	Fungi	*Talaromyces*	0.025	0.328	43
	Fungi	*Dothideomycetes*	0.028	0.378	39
	Fungi	*Orpinomyces*	0.008	0.367	27
	Fungi	*Pleosporales*	0.006	0.316	26
	Fungi	*Dothideomycetes*	0.005	0.366	25
S4	Fungi	*Sordariomycetes*	0.704	0.399	176
	Fungi	Unidentified	0.037	0.281	69
	Fungi	*Mycosphaerella*	0.141	0.323	68
	Fungi	*Cladophialophora*	0.046	0.310	56
	Fungi	*Sordariomycetes*	0.031	0.330	56
	Fungi	*Talaromyces*	0.030	0.289	42
	Bacteria	*Desulfobulbaceae*	0.059	0.255	41
	Fungi	*Nigrospora*	0.025	0.297	39
	Fungi	*Ascomycota*	0.027	0.298	38
	Fungi	*Dothideomycetes*	0.014	0.325	36
BS	Fungi	Unidentified	0.261	0.279	75
	Bacteria	*Bacteroidales*	0.218	0.247	75
	Archaea	*Halobacteriaceae*	0.149	0.272	45
	Fungi	*Tremellomycetes*	0.194	0.317	42
	Fungi	*Sordariomycetes*	0.108	0.283	42
	Fungi	*Nigrospora*	0.094	0.274	38
	Fungi	Unidentified	0.027	0.280	36
	Fungi	*Onygenaceae*	0.136	0.308	34
	Fungi	*Ascomycota*	0.118	0.286	32
	Bacteria	*Flammeovirgaceae*	0.089	0.154	32
SS1	Fungi	Unidentified	0.828	0.399	405
	Fungi	*Sordariomycetes*	0.059	0.325	47
	Fungi	Unidentified	0.211	0.348	40
	Fungi	*Saccharomycetales*	0.024	0.323	40
	Fungi	*Ascomycota*	0.056	0.337	35
	Fungi	*Trichosphaeriales*	0.060	0.337	32
	Archaea	*Cenarchaeaceae*	0.006	0.319	28
	Fungi	*Harknessia*	0.014	0.322	27
	Fungi	*Massarina*	0.010	0.323	22
	Archaea	YLA114	0.004	0.318	22
SS2	Fungi	Unidentified	0.168	0.353	72
	Bacteria	*Chromatiales*	0.252	0.306	62
	Fungi	*Pluteus*	0.192	0.386	60
	Fungi	Unidentified	0.115	0.360	60
	Fungi	*Harknessia*	0.061	0.370	53
	Fungi	*Dothideomycetes*	0.041	0.367	47
	Bacteria	*Candidatus Entotheonella*	0.109	0.301	45
	Fungi	Unidentified	0.082	0.368	45
	Fungi	*Ascomycota*	0.052	0.347	42
	Archaea	*Candidatus Nitrososphaera*	0.070	0.292	37
SS3	Fungi	*Antrodia*	0.315	0.341	88
	Bacteria	*Rhizobiales*	0.296	0.284	80
	Fungi	Unidentified	0.329	0.338	67
	Fungi	*Ascomycota*	0.035	0.291	49
	Fungi	*Mycosphaerellaceae*	0.049	0.303	43
	Fungi	Unidentified	0.048	0.304	43
	Fungi	*Saccharomyces*	0.046	0.302	38
	Fungi	*Pleosporales*	0.052	0.301	37
	Fungi	Unidentified	0.049	0.301	35
	Fungi	*Myrothecium*	0.039	0.299	32
SS4	Bacteria	*Chloroflexi*	0.410	0.246	120
	Fungi	Unidentified	0.385	0.292	65
	Fungi	Unidentified	0.048	0.262	55
	Fungi	*Ascomycota*	0.047	0.261	52
	Fungi	*Hypocreales*	0.037	0.261	51
	Fungi	Unidentified	0.182	0.277	47
	Fungi	*Ascomycota*	0.027	0.259	44
	Fungi	*Penicillium*	0.020	0.256	39
	Fungi	*Stictidaceae*	0.018	0.255	38
	Fungi	*Pleosporales*	0.076	0.272	33
BSS	Bacteria	*Myxococcales*	0.249	0.266	82
	Fungi	*Ascomycota*	0.145	0.293	82
	Fungi	Unidentified	0.461	0.323	75
	Fungi	*Saccharomycetaceae*	0.057	0.282	68
	Fungi	*Saccharomycetales*	0.052	0.286	64
	Fungi	*Ascomycota*	0.037	0.284	55
	Fungi	*Dothideomycetes*	0.037	0.283	53
	Fungi	*Ascomycota*	0.037	0.278	50
	Fungi	*Ascomycota*	0.011	0.269	40
	Fungi	*Talaromyces*	0.019	0.280	37
D1	Fungi	Unidentified	0.816	0.452	320
	Fungi	*Dothideomycetes*	0.260	0.376	92
	Bacteria	*Piscirickettsiaceae*	0.074	0.284	45
	Fungi	*Sordariomycetes*	0.017	0.355	42
	Fungi	*Sordariomycetes*	0.066	0.351	39
	Fungi	*Kotlabaea*	0.029	0.348	35
	Fungi	*Acremonium*	0.007	0.271	31
	Fungi	Unidentified	0.010	0.346	28
	Fungi	*Onygenaceae*	0.008	0.345	25
	Archaea	*Cenarchaeaceae*	0.001	0.262	25
D2	Fungi	*Nigrospora*	0.685	0.442	105
	Archaea	*Cenarchaeaceae*	0.267	0.383	49
	Bacteria	*Deltaproteobacteria*	0.172	0.359	46
	Archaea	*Crenarchaeota*	0.124	0.326	34
	Fungi	Unidentified	0.166	0.257	30
	Fungi	Unidentified	0.018	0.315	21
	Archaea	*Crenarchaeota*	0.007	0.331	21
	Bacteria	*Fulvivirga*	0.066	0.274	19
	Archaea	*Crenarchaeota*	0.033	0.339	19
	Archaea	*Crenarchaeota*	0.037	0.343	18
D3	Fungi	*Hypocreales*	0.264	0.283	92
	Fungi	*Abundisporus*	0.178	0.274	88
	Fungi	*Teratosphaeriaceae*	0.154	0.272	86
	Bacteria	*Desulfobacteraceae*	0.172	0.218	67
	Fungi	*Ascomycota*	0.033	0.260	50
	Bacteria	Unidentified	0.163	0.211	45
	Fungi	*Botryosphaeria*	0.224	0.255	41
	Fungi	*Diatrypaceae*	0.018	0.258	38
	Bacteria	*Chloroflexi*	0.069	0.253	32
	Fungi	*Dothideomycetes*	0.017	0.244	31
D4	Fungi	*Pleosporaceae*	0.522	0.319	141
	Fungi	Unidentified	0.109	0.303	81
	Fungi	*Hypocreales*	0.060	0.297	73
	Fungi	Basidiomycota	0.046	0.295	64
	Fungi	Unidentified	0.044	0.295	64
	Fungi	Unidentified	0.044	0.294	60
	Bacteria	*Hyphomonadaceae*	0.227	0.197	34
	Fungi	*Sordariomycetes*	0.039	0.286	33
	Fungi	*Phyllosticta*	0.065	0.272	29
	Fungi	*Abundisporus*	0.007	0.275	26
BD	Fungi	*Ascomycota*	0.859	0.468	428
	Fungi	*Teratosphaeriaceae*	0.041	0.267	87
	Archaea	*Nitrosopumilus*	0.040	0.286	37
	Archaea	*Cenarchaeaceae*	0.033	0.281	37
	Bacteria	*Piscirickettsiaceae*	0.047	0.238	36
	Bacteria	*Candidatus Portiera*	0.057	0.273	35
	Archaea	*Cenarchaeaceae*	0.044	0.241	34
	Bacteria	*Desulfuromonadales*	0.010	0.280	33
	Fungi	*Phyllosticta*	0.026	0.332	32
	Archaea	*Parvarchaea*	0.022	0.223	30

The lowest taxonomic resolution of the hub is shown.

### Burrow sediment environment and correlation with community composition

‘Depth’ and ‘Fraction’ had a significant effect on sediment biochemistry (PERMANOVA, *P* = 0.001 and *P* = 0.006, respectively; Supplementary Tables S8–10), and each ‘Depth’ was significantly different to the others (p-pht, *P* < 0.05). Significant effects (ANOVA) of ‘Fraction’ on POC, nitrate, sulphate and silicate and ‘Depth’ on PIC, sulphate and nitrite are reported in Table S11 (see also Supplementary File 3). ‘Depth’ effects on grain size are reported in Supplementary Table S12 and Supplementary Fig. S7.

**Figure 4:**
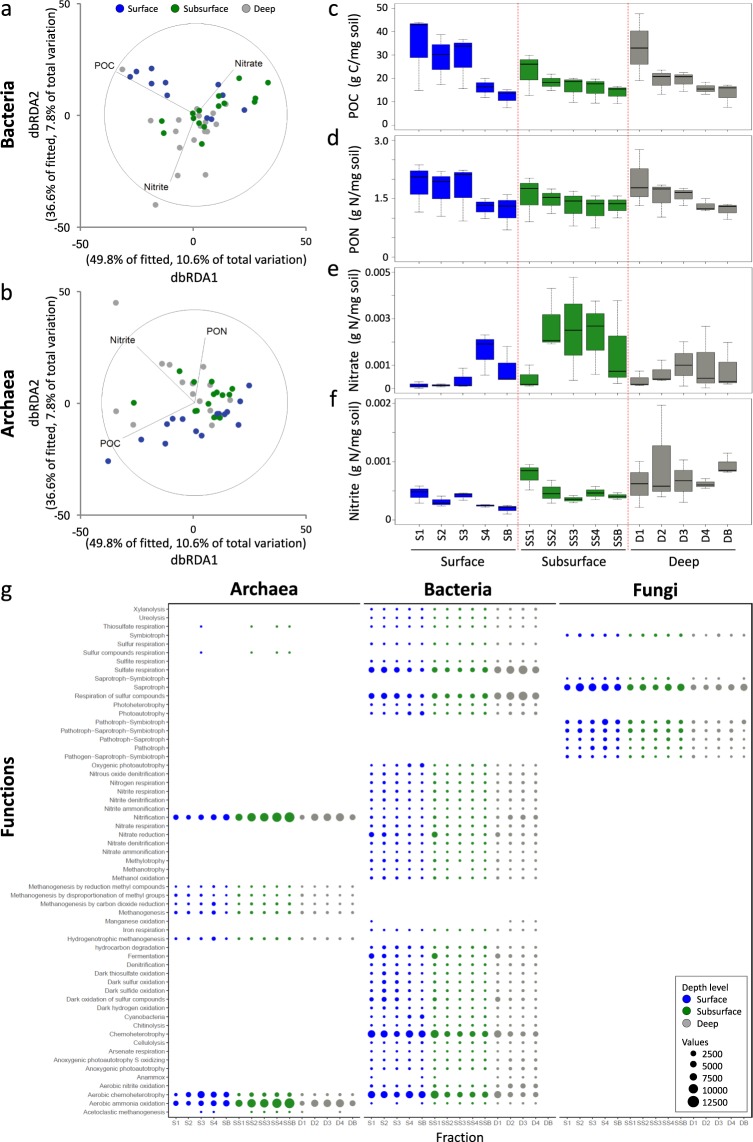
Distance Based Redundancy Analysis (db-RDA) of bacterial (**a**) and archaeal (**b**) communities highlighting the most significant environmental variables explaining the microbiome assembly. (**c–f** ) Variation of environmental parameters at each ‘Depth’ across ‘Fraction’ (POC, PON, Nitrate and Nitrite respectively). (**g**) Functional prediction of bacterial, archaeal and fungal communities at each ‘Depth’ across ‘Fraction’.

Bacterial beta diversity was significantly correlated with POC, nitrite and nitrate (DistLM, AICc = 342.46, R^2^ = 0.21; Fig. 4, Supplementary Table S13). Archaeal beta diversity was also significantly correlated with POC and nitrate, but additionally with PON (DistLM, AICc = 325.04, R^2^ = 0.26; Fig. 4, Supplementary Table S13). No variables correlated with fungal beta diversity (Supplementary Table S13).

### Functional groups

Bacterial, archaeal and fungal OTUs were assigned to predicted functional groups (Fig. 4g). A significant interaction of ‘Depth × Fraction’ was observed on bacterial OTU functional group assignment (PERMANOVA, F_8,80_ = 2.06, *P* = 0.001; Supplementary Fig. S8, Supplementary Table S14, see Supplementary File 4 for results of SIMPER). Aerobic chemoheterotrophs were abundant in all surface fractions and decreased in abundance away from the burrow wall in the subsurface and deep. Methanotrophic bacteria and those involved in fermentation were more abundant in Fraction 1 in the surface and deep and decreased toward bulk sediment at each ‘Depth’. OTUs assigned to the decomposition of plant matter, such as chitinolysis, cellulolysis and xylanolysis, were more abundant in the subsurface and deep, but not in Fraction 1, i.e., at the burrow wall, at these depths. *Cyanobacteria* and other photoautotrophs were observed to increase in abundance away from the burrow in the surface. The number of OTUs assigned to nitrate reduction were highest at the burrow wall (Fraction 1) in the surface, subsurface and deep.

A significant interaction of ‘Depth × Fraction’ was observed on archaeal OTU functional group assignment (PERMANOVA, F_8,108_ = 2.44, *P* = 0.001; Supplementary Fig. S8, Supplementary Table S14, see Supplementary File 4 for results of SIMPER), with the majority being assigned to the functions of nitrification, aerobic ammonia oxidation and aerobic chemoheterotrophy. Chemoheterotrophs and OTUs involved in methanogenesis were most abundant in the surface. OTUs involved in nitrification increased in abundance away from the burrow wall at every depth, being least abundant in bulk deep sediment.

A significant effect of ‘Depth’ was observed on fungal OTU functional group assignment (PERMANOVA, F_2,95_ = 3.59, *P* = 0.0015; Supplementary Fig. S8, Supplementary Table S14, see Supplementary File 4 for results of SIMPER), with deep sediment significantly differing from surface and subsurface sediment (p-pht, *P* < 0.05). The majority of fungal OTUs were classified as saprotrophic. In the surface, saprotrophs had the lowest abundance in Fraction 1 (i.e., at the immediate burrow entrance).

## Discussion

Acting as organic matter traps, burrows belonging to benthic herbivores of mangroves fuel microbial activity, promote niche diversification and habitat heterogeneity. As of yet we know little about mangrove sediment fungi other than their high diversity^23,24^, here we show that in these heterogeneous zones, fungi invariably play the major role in microbial network interactions from the internal burrow surfaces to the undisturbed sediments far from the burrow. Despite the varied sediment conditions from the burrow wall towards the bulk, and from the surface to the deep, the importance of fungi in the inter-kingdom networks was notable; fungal nodes were highly connected in every fraction of sediment (with the exception of deep bulk sediment), revealing a persistent central role of fungi in shaping the structure of the network (Table 1). In particular, the relationships among betweenness centrality and degree of connection (Fig. 3b) show that fungi persistently formed the keystone nodes essential for the interactome topology having, simultaneously, the highest levels of both of these network features. On the contrary, archaea and bacteria retained a high betweenness centrality but had a lower level of degree of connection revealing only a local effect on the overall topology of the network. These results are corroborated by other studies that observed fungi, as keystone species, to stabilize the network properties in other complex systems such as host-microbiome interactions^25^. Moreover, fungi showed a constant significant radiality compared to archaea and bacteria, which can be interpreted as a higher probability of an OTU to be functionally relevant for several other OTUs; eigenvector, that allows an immediate and informative evaluation of the interacting relevance of the OTU with the rest of the network, and the number of direct edges, indicating how the influence of each fungal node can extend to the rest of the network. Many studies have described fungi as superhighways, supporting their central role, that can enhance below ground communication, increasing the networking capabilities of the sediment microbiome and also networking with plants^26^. Certain members can establish long hyphae that boost the growth of microbial biofilms that are involved in the protection of the hyphae itself and in enhancing fungal communication^27^.

*N*. *africanum* is a highly efficient herbivore that removes up to 79% of mangrove ground litter^28,29^, storing more than half of this in their burrows^30^ allowing fungi to proliferate thereby increasing the palatability and nutritional value of mangrove litter^31^ (Supplementary File 5). Acting as hotspots of microbial activity, burrow walls were significantly enriched in POC and, correspondingly, higher numbers of bacteria, archaea and fungi. While POC availability was found to be a significant driver of bacterial and archaeal community composition, instead fungal community composition was unaffected. This may be explained by the broad metabolic plasticity of fungi^32^ and their dominance in the decomposition role in intertidal ecosystems^10,14^. At all depths, saprotrophic fungal nodes key to the microbial network were detected (e.g. the genus *Pluteus*) and fungi were the dominant network components throughout burrow sediment. By accumulating leaves in the walls of their burrows, *N*. *africanum* can facilitate and enrich the sediment fungal community with that of the leaves. Recently, the ability of fungi in the phyllosphere of the fallen leaves in a temperate beech forest to nurture the soil was demonstrated, by supplying a constant input of new fungal strains that establish in the forest litter and are involved in the litter decomposition process^33^. Even though we have not evaluated this aspect in the present study, we speculate that the burial of leaves by *N*. *africanum* can provide a constant input of fungal species to the burrow sediment, contributing to support the centrality of fungal taxa that shape the interactions and the assemblage of the entire sediment microbiome.

Although burrowing allows oxygen to penetrate into subsurface sediment, this oxygen is rapidly consumed by sediment microorganisms and organic-rich sediments characteristically have narrow zones of oxygen available as an electron acceptor. Indeed, anaerobic taxa were detected in the first layer of the burrow wall. Cellulose degradation in anaerobic sediment requires the interaction of microorganisms with diverse metabolism, with 5–10% degraded under anaerobic conditions by cellulose fermenting microbes to CH_4_, CO_2_ and H_2_O^34^. Fungi may have an important role in decomposition of organic matter in anoxic sediment that provide substrates to fermentative bacteria, such as the anaerobic bacterium *Pelobacteraceae*, which feed the archaeal methanogenic community^35^. Moreover, saprotrophic fungi from the *Basidiomycetes* are known to produce methane under oxic conditions^36^, promoting co-occurrence with methanotrophic microbes. While this study did not aim to resolve the steep gradients of carbon flow and electron acceptors in sediment, we detected a larger scale pattern around the burrow.

Burrows of intertidal benthic fauna are also hotspots of organic nitrogen, enhancing nitrogen cycling processes such as nitrification and denitrification due to the increase in oxic sediment zones created by burrow walls^37,38^. Fungi are predominantly aerobic heterotrophs, however they are also metabolically capable of utilizing nitrate and/or nitrite, thus they are key players in anaerobic denitrification in intertidal sediments with low oxygen availability^39^. While denitrifying bacteria are obligate anaerobes and dominate under strongly reducing sediment conditions, denitrifying fungi can make use of sub-oxic conditions (300–900 uM O_2_^39,40^). Denitrification by fungi under anaerobic conditions has particular ecological impacts, since rather than producing N_2_ as the final respiratory product, as do bacteria, the process ends with nitrous oxide, which is a significant greenhouse gas^41^. One of the functions to which bacteria were assigned in our study was nitrous oxide respiration, which may indicate a positive interaction between fungi and bacteria in *N*. *africanum* burrow sediment. Ingesting double the amount of litter they are able to assimilate, sesarmid crabs deposit highly nitrogen-rich faeces^42^. The availability of nitrate was a significant driver of the bacterial community, while PON was a significant driver of archaeal community composition. Nitrite influenced both archaeal and bacterial communities, while fungi were independent of both nitrite and nitrate. Due to the low availability of nitrate at the burrow wall (which increased away from the burrow) at every depth, we hypothesise that nitrate is rapidly consumed by the burrow wall microbiome and/or readily released into burrow water and flushed out at high tide^43^.

The high microbial community variability and turnover we observed in different sediment fractions, but also the patterns of sharing and abundance of certain taxa, may be explained by the large volume of sediment excavated from burrows by the host onto surface sediment around the burrow (sesarmid crabs are estimated to excavate in the range of 80–210 cm^3^ m^−2^ d^−1^ ^30^). Fungal diversity was higher in surface sediment unaffected by excavated material (in particular the orders *Diaporthales*, *Sordariomycetes* and *Agaricomycetes*), as was the abundance of photosynthetic bacteria such as *Cyanobacteria* and purple non-sulphur bacteria. In sediment closest to the burrow funnel and on the surrounding surface, we detected a number of sulphur compound reducing bacteria, indicating sulphur-compound rich and predominantly anaerobic sediment. This, along with the occurrence of similar taxa in Fraction 1 of the subsurface and surface of the burrow, can be explained by uni-directional excavation of burrow sediment, and thus microbes, and burrow maintenance. We detected a larger number of microbial OTUs to be shared between the surface, subsurface and deep sediment in the sediment adjacent to the burrow wall. Overall, however, deep sediment was found to have more unique OTUs which were not shared with other depth levels. Indeed, the deeper parts of *N*. *africanum* burrows are known to be highly stable over generations but the burrow openings are repeatedly repaired after high tides^44^, which would identify the absence of sediment mixing from the deep and the occurrence of a larger number of unique microbial OTUs at that depth. In a recent study of the smaller mangrove fiddler crab, which displays burrow plugging behaviour at high tide, sulphate reducing taxa were less abundant and aerobic taxa were more prevalent in sediment immediately around the burrow funnel^45^. This result may reflect the different behaviours of burrow hosts. Indeed, there was no indication of augmentation of oxygen through burrow sediment in this study.

Environmental variability guides the assembly of sediment bacteria and archaea by creating a halo of sequential physio-chemical characteristics around macrofaunal burrows, but here we suggest that key species detected by the study of the interactome also have an important effect on community assembly by driving co-occurrence patterns among microbial species. Understanding this complex relationship could be of pivotal importance in the comprehension of ecosystem functioning and restoration, especially for intertidal systems^46^. Combined, environmental factors and microbial species interactions can create new niches that boost microbial community diversity, stability and resilience through functional redundance^47,48^. For example, in riverine sediment contaminated with polycyclic aromatic hydrocarbons microbial co-occurrence patterns, interactions and key species varied with the extent of environmental contamination, thus demonstrating the combination of both the environmental factors and key network species in shaping sediment microbial communities^49^ and in this case revealing important microbial species for effective restoration/rehabilitation action. In other systems, such as grassland, fungal networks were found to be more resistant to changing environmental conditions in the form of drought stress than bacterial networks^50^, indicating that fungi may be more resilient than bacteria under environmental variability which could be essential for ecosystem resilience.

## Conclusions

Through their bioturbation activities and trapping mangrove leaves and organic matter inside their burrows, benthic fauna increase habitat heterogeneity and functional microbial diversity. At all the sediments depths and radial fractions that we investigated, the large proportion of the individuated keystone taxa were fungi (Fig. 3; Table 1), and only few archaea and bacteria provided central roles, but without consistent patterns. Our results indicate the synergistic contribution of fungi and environmental variability in structuring the microbial communities and their interactome throughout intertidal sediment. Since microbial networking has consequences on rates of organic matter decomposition and nutrient and blue carbon cycling processes, we highlight the necessity of a wholly inclusive approach in which the picture of the fungal microbiome and its role in the functional interactome cannot be overlooked.

## Materials and Methods

### Study site, study species, and sampling design

Sampling was carried out during April 2016 in a mixed *Avicennia marina* and *Rhizophora mucronata* riverine mangrove stand at the mouth of the temperate Mngazana estuary, South Africa (31°42′S, 29°25E). Sampling was performed 1 h before low tide, when burrows were uncovered by water, during the period of Spring Tide. We randomly sampled eight similar sized active *N*. *africanum* burrows along a 400 m transect, distinguished by fresh excavations and observation of the animal inside the burrow (Fig. 1a,b). Sediment sampling comprised of five sediment fractions along a horizontal gradient at the burrow wall (0–1 cm), mid-way along the excavated burrow content (13–14 cm), at the distal point of the excavated burrow content (26–27 cm) and a point away from the burrow (39–40 cm), which was repeated at three depths (surface: 0–1 cm, subsurface: 1–2 cm and deep: 20–21 cm; Fig. 1c). Bulk sediment was sampled at the three depths, >3 m away from the burrow and any visible root or pneumatophore or any other sign of bioturbation. From each burrow, we took 15 samples (total of 120 samples) stored on ice in the field and frozen at −20 °C within 3 h of collection.

### DNA sequencing, metabarcoding and geochemical analysis

DNA was extracted from a 0.4 g sub-sample of each sediment sample using the PowerSoil Total DNA Isolation Kit (MoBio Inc., CA, USA) following the manufacturer’s instructions. To identify bacterial and archaeal community composition we targeted the V4-V5 hypervariable region of the 16S rRNA gene using the primer pairs 341F-785R and 519F-806R respectively^51^. To identify fungal community composition, we amplified the ITS2 region of the internal transcribed spacer region using the primer pair ITS3-ITS4^52,53^. All libraries were prepared using the 96 Nextera XT Index Kit (Illumina®) and sequenced using the Illumina® MiSeq platform with pair-end sequencing at the Bioscience Core Lab, King Abdullah University of Science and Technology. Details on raw read processing are provided in the Supplementary File 1. QIIME was used to assign taxonomy, using the Greengenes database for bacteria and archaea and the UNITE database for fungi^54,55^. Copies of the 16S small-subunit rRNA gene for bacteria and archaea and the ITS region for fungi were quantified following the qPCR protocol described by Fierer and Jackson^56^ using the primer pairs Eub338-Eub518, Arc931F-Arc1100R and ITS1F-5.8 s for bacteria, archaea and fungi, respectively (see Supplementary File 1 for detail).

All geochemical, metal and grain size analyses of the sediment were performed in GEOMAR (Kiel, Germany). We obtained data on pH, particulate organic carbon (POC), particulate organic nitrogen (PON), particulate inorganic carbon (PIC), particulate inorganic nitrogen (PIN), nitrate, nitrite, silicate, phosphate, sulphate, chloride, the metals U, Pb, Al, Mn, Fe, Co, Ti, Ni, V, and Cr (see Supplementary File 1 for detail).

### Data analysis

All statistical tests were performed using PRIMER v. 6.1, PERMANOVA + for PRIMER routines^57^ and R software 3.4.1^58^. Homogeneity of multivariate dispersion for each factor was tested using the distant-based test PERMDISP prior to PERMANOVA analyses. For all analyses, our explanatory variables were the categorical factors (fixed, orthogonal) ‘Depth’ (3 levels: surface (0–1 cm), subsurface (1–2 cm), deep (20–21 cm)) and ‘Fraction’ (5 levels corresponding to 5 distances away from the burrow: 1 (0–1 cm), 2 (13–14 cm), 3 (26–27 cm), 4 (39–40 cm) and bulk (>3 meters)) (Fig. 1c).

We tested differences in beta-diversity of bacteria, archaea and fungi amongst ‘Depth’ and ‘Fraction’ using a multivariate generalized linear model (GLM) with a negative binomial error distribution, in the R package “mvabund”^59^. In this test, and those following, we treated the burrow as a random effect by using the function ‘manyany’ where it is possible to specify the random factor. We used a GLM to test differences in bacterial, archaeal and fungal gene copies, using a log transformation for normality, among ‘Depth’ and ‘Fraction’. OTU assemblages across ‘Depth’ were explored using Canonical Analysis of Principal coordinates (CAP^60^). The Shannon diversity index and OTU richness were calculated using the function ‘diverse’ in PRIMER (v. 6.1) and 2-way PERMANOVA was used to test differences in diversity and richness among the factors ‘Depth’ and ‘Fraction’.

Ternary plots, using the mean relative abundances of bacteria, archaea and fungi OTUs in surface, subsurface and deep sediment, were created using the R package “ggtern”^61^ to examine the number of OTUs shared amongst different depths. The Sloan model^62^ was used to test the homogenizing effect of sesarmid activity on the bacterial, archaeal and fungal communities in each ‘Fraction’ of the burrow. We determined if the structure of a given community fits a neutral assembly model where the abundances of taxa are driven by dispersal from a source community. For example, to test whether there is significant vertical mixing downwards, we examined if the abundances of the taxa in the subsurface or deep communities are driven by dispersal from the surface or subsurface communities, respectively. To identify taxa that were discriminately more abundant in each ‘Fraction’ at each ‘Depth’ (Wilcoxon *P* value: 0.05, LDA > 2), we used Linear discriminant analysis effect size (LEfSe, www.huttenhower.sph.harvard.edu/galaxy/) following Segata *et al*.^63^.

To identify co-existing or mutually exclusive OTUs amongst bacteria, archaea and fungi, an inter-kingdom co-occurrence network was built following Agler *et al*.^64^ using the routine CoNet in Cytoscape 3.2.1^65^. Cytoscape was used to calculate the network topological parameters^66^ and Gephi 1.9 to compute network modularity and visualization^67,68^. Network topological coefficients were calculated using the cytoscape plug-in Centiscape^69^ and Network analyser^66^. Further details on the network analysis are provided in Supplementary File 1. Network centrality measures were compared across ‘Depth’ and ‘Fraction’ adding the explanatory variable ‘Kingdom’ (3 levels: archaea, bacteria, fungi*;* fixed and orthogonal) and 3-way analysis of variance (ANOVA) was used to test the effect of ‘Kingdom’, ‘Depth’ and ‘Fraction’ on degree of connection and average path length. A GLM with a quasibinomial error distribution was used to test the effect of ‘Kingdom’, ‘Depth’ and ‘Fraction’ on closeness, betweenness centrality measures, radiality, eigenvector and number of directed edges using the R package “MASS”^58,70^.

Keystone species were analysed by testing the relationship between degree of connection and betweenness centrality following de Vries^50^ using a Generalized Additive Model included in the r packge mgcv^71^. Keystone species were detected by combining the highest level of three centrality measures: degree of connection, closeness centrality and betweenness centrality following Berry and Widder^72^.

After testing for multi-collinearity (see Supplementary File 1 for further detail), 2-way PERMANOVA (9999 permutations, Euclidean distance) was used to test differences in biochemistry, metals and grain size for ‘Depth’ and ‘Fraction’. The contribution of different geochemical variables, metals and grain size classes to dissimilarity between each ‘Depth’ and ‘Fraction’ was assessed by SIMPER analysis. Grain size frequencies were calculated using the R package “G2sd”^73^. Distance-based multivariate analysis for a linear model^57^ was used to determine the geochemical variables, metals and grain size classes that significantly explained the variation community composition amongst ‘Kingdom’ (using the corrected Akaike information criterion (AICc) to test significance^74^). Differences in sediment phi were tested for ‘Fraction’ and ‘Depth’ with a 2-way ANOVA.

The FAPROTAX database was used to assign bacterial and archaeal OTUs to known metabolic or ecological functions (http://www.zoology.ubc.ca/louca/FAPROTAX75). We used the annotation tool FUNGuild v1.0^9^ to assign functional guild, in terms of trophic mode, to fungal OTUs. 2-way PERMANOVA was used to test differences in functional group assignment amongst ‘Depth’ and ‘Fraction’ for each ‘Kingdom’. The contribution of functional group assignment to dissimilarity was assessed by SIMPER analysis.

## Supplementary information

Supplementary file 1

Supplementary file 5

Supplementary file 2

Supplementary file 3

Supplementary file 4

## Data Availability

The datasets generated during the study are available in the NCBI SRA repository under the BioProject ID: PRJNA415044^75^.
