# Supplementary material for: The role of fungi in heterogeneous sediment microbial networks
Source: Sci Rep. 2019 May 17;9:7537. doi: 10.1038/s41598-019-43980-3 (PMC6525233; doi:10.1038/s41598-019-43980-3)
Supplement: Supplementary file 1 [file 41598_2019_43980_MOESM1_ESM.pdf]

Supplementary File 1: Supplementary methods, supplementary results, supplementary Figures and Tables

The role of fungi in heterogeneous sediment microbial networks

Jenny Marie Booth[†], Marco Fusi[†,*], Ramona Marasco, Grégoire Michoud, Stilianos Fodelianakis, Giuseppe Merlino, Daniele Daffonchio[*]

King Abdullah University of Science and Technology, Red Sea Research Center, Thuwal 23955-6900, Saudi Arabia

[†]Equally contributed.
[*]Corresponding authors: marco.fusi@kaust.edu.sa; daniele.daffonchio@kaust.edu.sa

Supplementary methods

Study site and species

At the southern limit of mangrove distribution, Mngazana has an annual average temperature of 18°C and annual average precipitation of 120 mm. *Neosarmatium africanum* is highly abundant in the Mngazana mangrove, which is estimated to support 6 million individuals in 150 ha of mangrove with population densities of approximately 0.2 to 10 m^{-2} [1]. Sesarmid crabs are referred to as 'ecosystem engineers'[2], digging large complex and interlinked burrows that can reach depths of 2 m in the intertidal belt[3]. *N. africanum* feeds on sediment detritus and fallen mangrove leaves[4] and is estimated to consume 44% of mangrove leaf fall (0.78 g m^{-2} d^{-1}) in the Mngazana forest[1], accumulating large stocks of organic matter in their burrows[5]. We chose *N. africanum*, belonging to the Sesarmidae family, as our study species because sesarmid crabs are amongst the most widely distributed macrofaunal bioturbators in indo-pacific mangrove forests[6] (Fig. 1a). The ecology of the burrow inhabitant is an important consideration when assessing the impact of bioturbation. *N. africanum* typically exit their burrows from one side, which is usually dictated by the formation of a hood over the burrow (Fig. 1B). In this direction, the crabs excavate their burrows depositing a large amount of debris up to a distance of >30 cm (observed in this study) from the burrow entrance.

Raw read processing

For bacteria and fungi, raw forward and reverse reads for each sample were assembled into paired-end reads considering a minimum overlap of 50 nucleotides and maximum of one mismatch within the region using the fastq-join algorithm (https://code.google.com/p/ea-utils/wiki/FastqJoin). For archaea, forward reads only were used due to insufficient quality of the reverse reads. Following data quality checks, we discarded reads with mean quality below 20. Sequence dereplication was performed using UPARSE v8 and QIIME v1.8 software. Reference chimera detection was carried out using the "Gold" database in the Broad Microbiome Utilities for bacteria and archaea. QIIME was used to assign taxonomy, using the Greengenes database for bacteria and archaea and the UNITE database for fungi[7,8]. We excluded samples with coverage below 90% prior to generating a separate OTU table for bacteria and archaea (⩾97% sequence similarity of 16S rRNA gene sequences) and fungi (⩾97% sequence similarity of ITS2). All analyses were performed on the filtered OTU tables (reads filtered to 0.01%).

Quantification of the bacterial, archaeal and fungal communities associated with *N. africanum* burrows

Fragments were amplified from the total genomic DNA isolated from sediment samples as described above. Quantitative PCR reactions were performed in a Rotor-Gene Q thermocycler (Qiagen) using the GoTaq® qPCR Sybr Green Master Mix (Promega). Samples were first quantified using the Qubit dsDNA BR Assay Kit prior to dilution of each to 2 ng/µl to be used as template DNA. In cases where sample concentration was too low for dilution, samples were used undiluted. PCR reactions were performed in a volume of 15 µl, containing 1X GoTaq® Master Mix (with 2mM $MgCl_2$), 100 nM of each primer for bacteria and archaea and 400 nM of ITS1F and 5.8s for fungi, and 1.5 µl of template DNA. Quantitative PCR conditions for bacteria and archaea were: 95°C for 2 min, 45 cycles at 95°C for 15 s, 53°C (bacteria) or 64°C (archaea) for 20 s and 60°C for 20 s. At the end of the run, denaturation curves were obtained by performing re-naturation at 50°C for 180 s, followed by gradual denaturation for 91 cycles from 50°C to

95°C with an increase of 0.5°C/cycle every 5 s. qPCR conditions for fungi were: 95°C for 2 min, 45 cycles at 95°C for 40 s, 55°C for 40 s and 60°C for 60 s. Standard curves were created for each qPCR assay, with a series of dilutions ranging from 50 to $5x10^7$ copies/µl. All standards and samples were run in triplicates. Concentration in terms of copies/µl, for every sample was calculated from the standard curve. In all the qPCR assays run, R^2 varied between 0.99309 and 0.99908 and amplification efficiencies varied between 85% and 107% across the different qPCR assays performed.

Geochemical analysis

Grain size measurements were obtained according to Beuselinck[9]. Particulate carbon and nitrogen was analysed with an elemental analyser (Thermo Finnegan Flash EA1112). Removal by acidification of POC (Particulate Organic Carbon) and PON (Particulate Organic Nitrogen) (sulphurous acid (H_2SO_3) under vacuum for 24-48 h yielded PIC (Particulate Inorganic Carbon) and PIN (Particulate Inorganic Nitrogen) after subtraction from total carbon and nitrogen[10]. Nutrient analysis was performed by prior leaching of sediment with deonised water (following EPA protocol[11]). Nitrate, nitrite, silicate and phosphate were determined using auto-analyser techniques[12] (Seal AA3 instrument). Sulphate and chloride were obtained using ion chromatography[13] (Dionex). pH was measured using a Metrohm pH meter with an Orion-Thermofischer electrode. A microwave-assisted acid digestion procedure was used to dissolve sediments[14] for subsequent inductively coupled plasma mass spectrometry (ICP-MS) analysis. Total elemental concentrations of U, Pb, Al, Mn, Fe, Co, Ti, Ni, V, and Cr using an ICP-MS instrument were obtained (Element II, Thermo Fisher Scientific).

Data analysis

Network analysis. After removal of rare OTUs (less than 0.01% of sequences per sample), the network was constructed by combining Pearson and Spearman correlation coefficients with two other distance-based methods, specifically Bray-Curtis and Kullback-Leibler indices, as indicated in Faust and Raes[15]. The different measures that we used (Pearson, Spearman, Bray Curtis, Kullback-Leibler) capture different types of relationships, but they converge when thresholds are increased. Moreover, the specific four *P* values obtained for each edge were merged (using Fisher's method) and corrected for multiple testing (Benjamini-Hochberg correction). Edges with *P* values above a threshold of 0.05 were discarded. In order to calculate statistical significance of OTU co-occurrence/mutual exclusion, data from edge-specific permutation and bootstrap score distributions with 1000 iterations were first normalized, allowing us to acquire the similarity introduced by compositionality only. A *P* value was obtained using pooled variance to z-score the permutated null and bootstrap confidence[16]. We dissected the microbial networks for the explanatory variable 'Fraction' - 'Depth' interaction, calculating centrality measures such as: degree of connection (extent of taxon connection working as hub), closeness centrality (extent of influence of a node on the entire network) and betweenness centrality (extent of how much anode influence on the surrounding node in the network[17]). Furthermore, to detect the keystones species we computed the radiality, eigenvetor and number of directed nodes. We also measured the average path length (extent of the efficiency of the bacterial connection in a network). and the edge-betweenness centrality was used to analyse phylum-specific bridge-like connectors between two parts of a network (the removal of which may affect the connectivity between many pairs of nodes through the shortest paths between them[17]).

Geochemical analysis. We tested all geochemical variables and metals for multi-collinearity using the non-parametric Spearman correlation and Draftsman's plots on normalized data prior to analysis. In cases where variables had a correlation coefficient higher than 0.85, we retained the most appropriate and informative variables (chloride and the metals nickel and vanadium were removed from further analyses).
Functional assignment. The FAPROTAX database was used to assign bacteria and archaea OTUs to known metabolic or ecological functions (http://www.zoology.ubc.ca/louca/FAPROTAX[18]). This database maps a taxon to a function based on all cultured representatives within that taxon having exhibited the specific function. We acknowledge that this may be biased by its restriction to cultured representatives. Using this database, taxa with no cultured representatives cannot be functionally annotated

Supplementary Results

Quantitative PCR

We detected a significant effect of 'Depth' (ANOVA, $F_{2,104}$= 15.97, $P < 0.001$) and 'Fraction' (ANOVA, $F_{4,104}$= 8.84, $P < 0.001$) on the number of copies of bacteria per gram of sediment, decreasing from $4.13e^{+09}$ to $9.73e^{+08}$ copies in the bulk in surface sediment, from $2.33e^{+09}$ to $8.16e^{+08}$ copies in the bulk in subsurface sediment, and from $2.97e^{+09}$ to $2.07e^{+08}$ copies in the bulk in deep sediment. For archaea we detected a significant interaction between 'Depth' and 'Fraction' (ANOVA, $F_{8,105}$= 2.37, $P < 0.05$), with lower values than bacteria ranging from $7.40e^{+07}$ to $4.53e^{+07}$ copies in the bulk in surface sediment, from $3.45e^{+07}$ to $2.56e^{+07}$ copies in the bulk in subsurface sediment, and from $7.71e^{+07}$ to $4.55e^{+06}$ copies in the bulk in deep sediment. We observed the same significant interaction between 'Depth' and 'Factor' for fungi (ANOVA, $F_{8,104}$= 2.11, $P < 0.05$), with values ranging from $7.54e^{+07}$ to $5.96e^{+07}$ copies in the bulk in surface sediment, from $7.50e^{+07}$ to $6.67e^{+06}$ copies in the bulk in subsurface sediment, and from $4.41e^{+07}$ to $3.73e^{+05}$ copies in the bulk in deep sediment (Fig. 1 D-F).

Community diversity and richness

Bacterial alpha diversity and richness were consistently higher at every 'Depth' and in each 'Fraction' compared to those of archaea and fungi. No significant effect of 'Depth' or 'Fraction' was observed on either bacterial diversity or richness (PERMANOVA, $P > 0.05$ in both cases; Fig. S1, Table S3). A significant interaction of 'Depth' x 'Fraction' was observed on archaeal and fungal species diversity and richness (PERMANOVA, $P = 0.001$ and $P = 0.01$, respectively, for both diversity and richness; Fig. S1, Table S3). In the deep, archaeal alpha diversity differed among fractions, with higher diversity observed in Fraction 1 at the burrow wall (p-pht, $P < 0.05$; Table S4), but not in the surface and subsurface. Differences in archaeal richness were observed between each 'Fraction' at every 'Depth' (p-pht, $P < 0.05$; Table S5). For fungi, certain fractions in the surface and deep displayed significantly different diversity and richness (p-pht, $P < 0.05$; Table S4 and S5), while no differences among 'Fraction' were observed in the subsurface.

Community composition

Bacteria. Overall, four phyla were dominant across depth levels, with different overall contributions to community composition (Fig. S2). In the surface and subsurface, *Proteobacteria* and *Bacteroidetes* were dominant (Kruskall-Wallis test, $P < 0.05$, Table S6). *Chloroflexi* and *Cyanobacteria* were the next largest contributors to community composition in the surface, and

the former was also a dominant phylum in the subsurface. In the deep, *Proteobacteria* was the dominant phylum. Across all depths, the contribution of each phyla to community composition significantly differed between depths (Kruskall-Wallis test, $P < 0.05$ in all cases, Table S6). Changing community structure along a horizontal gradient was also observed (Fig. S2). In the surface, the *delta*-, *gamma*- and *epsilon*- divisions of *Proteobacteria* were more important in Fractions 1 to 3 (i.e. in the excavated burrow material, Kruskall-Wallis test, $P < 0.05$ in all cases, Table S7). Instead, the *epsilon-Proteobacteria* decreased away from the burrow wall in the subsurface, with the addition of an observed increase in *Actinobacteria* and *Gemmatimonadetes* towards bulk sediment (Kruskall-Wallis test, $P < 0.05$ in all cases, Table S7). A notable reduction in *epsilon-Proteobacteria* after Fraction 1 towards bulk sediment and increase in *Alphaproteobacteria* and *Acidobacteria* was observed in deep sediment (Kruskall-Wallis test, $P < 0.05$ in all cases, Table S7).

Archaea. The phyla *Halobacteria*, MCG, *Parvarchaea* and *Thaumarchaeota* were the dominant contributors to community composition across all fractions, with the contribution of *Halobacteria* and *Parvarchaea* significantly decreasing and the contribution of *Thaumarchaeota* significantly increasing in the deep (Kruskall-Wallis test, $P < 0.05$ in all cases, Table S6). In the surface, *Thermoplasmata* and MCG were more abundant in Fractions 1 to 3, while *Halobacteria* increased away from the burrow wall (Kruskall-Wallis test, $P < 0.05$ in all cases, Table S7). In the subsurface, *Halobacteria* had a significantly smaller contribution to community composition in Fraction 1, at the burrow wall, compared the other burrow fractions, while *Thermoplasmata* had a higher contribution in SS1 and SS2 compared to other fractions (Kruskall-Wallis test, $P < 0.05$ in all cases, Table S7). While in deep sediment, *Parvarchaea* had a clear higher dominance in Fraction 1 compared to the other fractions (Kruskall-Wallis test, $P < 0.05$ in all cases, Table S6).
Fungi. All sediment fractions were dominated by the phyla *Ascomycota* and *Basidiomycota* (Fig. S2), with a significant change in their contribution to community composition across depth (Kruskall Wallis, $P = 0.002$ and $P = 0.001$ respectively; Table S6).

Network topology
The centrality measures differed for each ‘Kingdom’ across each ‘Fraction’ at each ‘Depth’. In surface sediment, a significant effect of ‘Kingdom’ only was observed on degree of connection (ANOVA, $F_{2,3286} = 110.69$, $P < 0.001$; Fig. S5A, E, I), revealing a similar level of connection across ‘Fraction’, but with a higher connectivity of fungi compared to archaea and bacteria. In subsurface and deep sediment, a significant interaction of ‘Fraction × Kingdom’ was observed on degree of connection (respectively, ANOVA: $F_{2,3373}=3.847$, $P < 0.001$; $F_{4,3612}=4.307$, $P = < 0.001$; Fig. S5A, E, I), indicating a significant variation in connectivity within each ‘Kingdom’ for each ‘Fraction’. However, fungi showed a higher degree of connection in every ‘Fraction’ at every ‘Depth’ than bacteria and archaea, with the exception of deep bulk sediment.
A significant interaction of ‘Fraction × Kingdom’ was observed on average shortest path length in surface, subsurface and deep sediment (respectively, ANOVA: $F_{8,3286}= 71.51$, $P < 0.001$; $F_{8,3373}= 2.497$, $P < 0.05$; $F_{8,3612}= 6.998$, $P < 0.001$; Fig. S5B, F, L). In the surface and subsurface, the average path length increased towards the bulk sediment with a higher path length attributed to archaea and bacteria. In the deep, for all kingdoms, there was a general increase towards Fraction 3 and a subsequent decrease towards the bulk sediment. Average shortest path length was lowest for Fungi in Fraction 1 and fungi and bacteria in the deep bulk.

A significant interaction of 'Fraction × Kingdom' was observed on closeness centrality at all depths. In the surface (χ^2, deviance $_{8,3286}$ = 770.04, $P < 0.001$; Fig. S5C, G, M) an overall decrease toward the bulk was observed; bacteria showed a higher closeness centrality in the four burrow fractions than in the bulk, while instead fungi closeness centrality increased in the bulk. In the subsurface (χ^2, deviance $_{8,3373}$ = 725.01 $P < 0.01$), across the fractions, archaea showed higher variability especially in the second fraction and in the bulk sediment. In the deep (χ^2 deviance $_{8,3612}$ = 720.38, $P < 0.001$), fungi had the highest closeness centrality in Fraction 1, while bacteria and archaea had higher closeness centrality in the subsequent burrow fractions. In the bulk, bacteria and fungi had a higher closeness centrality than archaea.

At all three depths, across all fractions, fungi had a persistently higher betweenness centrality. In surface sediment, a significant interaction of 'Fraction × Kingdom' was observed on betweenness centrality (χ^2 deviance $_{8,3286}$ = 992.91, $P < 0.001$), showing a differential level of betweenness across 'Fraction' and 'Kingdom'. In subsurface sediment, a significant effect of 'Fraction' (χ^2 deviance $_{4,3383}$ = 790.85, $P < 0.01$) and 'Kingdom' (χ^2 deviance $_{2,3381}$ = 782.87, $P < 0.01$) with no significant interaction was observed (Fig. S5D, H, N). Similarly, in the deep a significant effect of both 'Fraction' (χ^2 deviance $_{4,3622}$ = 78.20, $P < 0.001$) and 'Kingdom' (χ^2 $_{2,3620}$ = 773.61, $P < 0.05$) was observed on betweenness centrality. Betweenness centrality was highest for fungi across every 'Fraction' in the surface, confirming the higher connectivity of fungi compared to bacteria and archaea.

Edge betweenness was highly variable across 'Depth' and 'Fraction' (Fig. S6). In particular fungi-fungi and archaea-archaea edge betweenness was lowest at the burrow wall and in all burrow fractions, but fungi-fungi increased in the deep bulk sediment. Although variable, a consistently higher value between fungi-bacteria and fungi-archaea was detected in each 'Depth' and 'Fraction', confirming the previous observation of a high centrality for fungi.

The network topological parameters highlight a specific network structure for each 'Depth' and 'Fraction' for each Kingdom. The clustering coefficient tended to be lower in the bulk sediment at each 'Depth', but in the burrow it was higher in the deep compared to the surface and subsurface (Fig. S4A). The clustering coefficient was consistently lower in the surface sediment across all fractions, and in the deep it increased from Fraction 1 to 4 and decreased again in the bulk (Fig. S4B). Centralization was highest in Fraction 1 at all depths, and in the deep it was also very high in the bulk (Fig. S4C). Overall, average path length was shortest in Fractions 1 and 2 at all depths and bulk sediment in the deep (Fig. S4D). The average number of neighbours was shortest around the burrow, in Fraction 1-3 in the surface and deep, and additionally in the deep bulk (Fig. S4E). A decrease in the number of nodes was observed along a gradient of Fraction 1 to bulk sediment in the surface; and overall this number was highest in Fraction 1 of the subsurface and deep and also in bulk deep sediment (Fig. S4F). Density was lowest in Fraction 1 at all depths and in the bulk of the deep sediment, showing an increasing trend towards bulk sediment in the surface and in the subsurface from Fraction 2; (Fig. S4G). Heterogeneity was highest in Fraction 1 at all depths, and also in bulk deep sediment (Fig. S4H). The number of interactions consistently decreased across all fractions in surface and subsurface sediment; in bulk sediment it was highest in the deep (Fig. S4I). Modularity was lowest in Fraction 1 of surface and subsurface sediment, whilst being highest in Fraction 1 of deep sediment (Fig. S4J).

Sediment environment and correlation with community composition

Mean geochemical (POC, PON, PIC, PIN, nitrate, nitrite, silicate, phosphate and sulphate), metal (U, Pb, Al, Mn Fe, Co, Ti and Cr) and grain size values are provided in Supplementary File 3.

No significant effects of either ‘Depth’ (PERMANOVA, $F_{2,30} = 0.36$, $P = 0.69$) or ‘Fraction’ (PERMANOVA, $F_{4,30} = 0.95$, $P = 0.2$) were found on sediment pH. No effects of either ‘Fraction’ or ‘Depth’ were observed on PON, PIN or phosphate ANOVA, $P > 0.05$). No significant effects of either ‘Depth’ or ‘Fraction’ were found on sediment metal content (PERMANOVA, $P > 0.05$ in both cases; Table S9).

A significant effect of ‘Depth’ was found on grain size (PERMANOVA, $P = 0.002$; Table S9, S12); deep sediment significantly differed from both surface and subsurface sediment (p-pht, $p < 0.05$ in both cases). This was corroborated by the significant effect of ‘Depth’ on sediment phi (ANOVA, $F_{2,30} = 5.75$, $p < 0.05$; Fig. S7). Bacterial, archaeal and fungal beta diversity were not correlated with sediment metal content. Certain sediment grain size classes were found to drive archaeal beta diversity only (1.88, 60.8, 0.83 and 4.86, DistLM, AICc = 326.1, $R^2 = 0.28$; Table S13).

Surface sediment differed from deep sediment predominantly in the content of particulate inorganic carbon (PIC), sulphate, particulate organic carbon (POC), silicate and particulate inorganic nitrogen (PIN) (SIMPER, cumulative 90.7%; Table S10). Surface and subsurface sediment differed predominantly in PIC, sulphate, POC, PIN and silicate (SIMPER, cumulative 95.5%; Table S10). Subsurface differed from deep sediment predominantly in silicate, POC, sulphate and phosphate (SIMPER, cumulative 92.4%; Table S9). On a horizontal gradient, Fraction 1 differed from Fractions 3, 4 and bulk (p-pht, $p < 0.05$). A total of 92% dissimilarity between Fraction 1 and bulk sediment was caused by variability in PIC, POC, sulphate PIN and silicate (SIMPER; Table S10). 91.5% dissimilarity between Fraction 1 and Fraction 3 was explained by variability in PIC, POC, sulphate, PIN and phosphate. A total of 92% dissimilarity between Fraction 1 and Fraction 4 was caused by variability in PIC, POC, PIN, silicate and phosphate.

Between the surface and deep, 60% of dissimilarity was contributed by grain size classes ranging from 31-58.2 μm (SIMPER; Table S13). Between the subsurface and deep, 70% of dissimilarity was contributed by grain size classes ranging from 29.6-53.2 μm (SIMPER; Table S12). Between the surface and subsurface, 69% of dissimilarity was contributed by the coarser grain size classes ranging from 40.6-58.2 μm (SIMPER; Table S13).

Functional group assignment

SIMPER analysis revealed that at least 50% of the dissimilarity in bacterial OTU functional assignment between each ‘Fraction’ and ‘Depth’ was attributed the functions sulphate respiration, respiration of sulphur compounds, chemoheterotrophy and aerobic chemoheterotrophy (Supplementary File 4).

SIMPER analysis revealed 48% of the dissimilarity in archaeal OTU functional assignment between the surface and deep sediment was attributed to the functions nitrate reduction, nitrate respiration, chemoheterotrophy and aerobic chemoheterotrophy; between the subsurface and deep, 51% was attributed to the functions nitrate reduction, nitrate respiration, nitrogen respiration, chemoheterotrophy and aerobic chemoheterotrophy; while between the surface and subsurface, 43% was attributed to methanogenesis by disproportionation of methyl groups, methanogenesis by CO_2 reduction with H_2, aerobic ammonia oxidation, nitrification and nitrate reduction (SIMPER, Supplementary File 4). Assignment of OTUs to the function methanogenesis by disproportionation of methyl groups caused at least 10% of dissimilarity amongst the bulk and Fractions 1, 2 and 3 and between Fractions 1 and Fractions 2, 3 and 4 and between Fraction 3 and Fraction 4 (SIMPER, Supplementary File 4).

At least 55% of dissimilarity in functional assignment of fungal OTUs between the deep and surface and the deep and subsurface was assignment of OTUs to the groups saprotroph and symbiotroph; while between the surface and subsurface, 56% was caused by assignment to the functions symbiotroph and pathotroph-symbiotroph (Supplementary File 4).

References

1. Emmerson, W. D. & Mcgwynne, L. E. Feeding and assimilation of mangrove leaves by the crab *Sesarma meinerti* de Man in relation to leaf-litter production in Mgazana , a warm-temperate southern African mangrove swamp. *J. Exp Mar. Biol. Ecol.* **157,** 41–53 (1992).
2. Kristensen, E. Mangrove crabs as ecosystem engineers; with emphasis on sediment processes. *J. Sea Res.* **59,** 30–43 (2008).
3. Berti, R., Cannicci, S., Fabbroni, S. & Innocenti, G. Notes on the structure and the use of *Neosarmatium meinerti* and *Cardisoma carnife*x burrows in a kenyan mangrove swamp (decapoda brachyura). *Ethol. Ecol. Evol.* **20,** 101–113 (2008).
4. Skov, M. W. & Hartnoll, R. G. Paradoxical selective feeding on a low-nutrient diet: Why do mangrove crabs eat leaves? *Oecologia* **131,** 1–7 (2002).
5. Andreetta, A. *et al.* Mangrove carbon sink. Do burrowing crabs contribute to sediment carbon storage? Evidence from a Kenyan mangrove system. *J. Sea Res.* **85,** 524–533 (2014).
6. Ragionieri, L., Fratini, S. & Schubart, C. D. Revision of the *Neosarmatium meinerti* species complex (Decapoda: Brachyura: Sesarmidae), with descriptions of three pseudocryptic Indo–West Pacific species. *Raffles Bull. Zool.* **60,** 71–87 (2012).
7. DeSantis, T. Z. *et al.* Greengenes, a chimera-checked 16S rRNA gene database and workbench compatible with ARB. *Appl. Environ. Microbiol.* **72,** 5069–5072 (2006).
8. Kõljalg, U. *et al.* Towards a unified paradigm for sequence-based identification of fungi. *Mol. Ecol.* **22,** 5271–5277 (2013).
9. Beuselinck, L. Grain-size analysis by laser diffractometry: comparison with the sieve-pipette method. *Catena* **32**,193–208 (1998).
10. Verardo, D. J., Froehlich, P. N. & McIntyre, A. Determination of organic carbon and nitrogen in marine sediments using the Carlo-Erba NA 1500 analyzer. *Deep. Res.* **37,** 157–165 (1990).
11. Plumb, R. *Procedures for handling and chemical analysis of sediment and water samples*. (State University of New York College at Buffalo Great Lakes Lab, 1981).
12. Grasshoff, K., Kremling, K. & Ehrhardt, M. *Methods of Seawater Analysis*. (Verlag Chemie, 1983).
13. Morales, J. A., De Graterol, L. S. & Mesa, J. Determination of chloride, sulfate and nitrate in groundwater samples by ion chromatography. *J. Chromatogr. A* **884,** 185–190 (2000).

14. Hassan, N. M., Rasmussen, P. E., Dabek-zlotorzynska, E., Celo, V. & Chen, H. Analysis of environmental samples using microwave-assisted acid digestion and inductively coupled plasma mass spectrometry: Maximizing total element recoveries. *Water, Air, Soil Pollution* **178**, 323–334 (2007).

15. Faust, K. & Raes, J. CoNet app: inference of biological association networks using Cytoscape. *F1000Research* **5,** 1519 (2016).

16. Barberán, A., Bates, S. T., Casamayor, E. O. & Fierer, N. Using network analysis to explore co-occurrence patterns in soil microbial communities. *ISME J.* **6,** 343–351 (2011).

17. Doncheva, N. T., Assenov, Y., Domingues, F. S. & Albrecht, M. Topological analysis and interactive visualization of biological networks and protein structures. *Nat. Protoc.* **7,** 670–685 (2012).

18. Louca, S., Parfrey, L.W., Doebeli, M. Decoupling function and taxonomy in the global ocean microbiome. *Science* **353**, 1272-1277 (2016).

Supplementary Figures and Tables

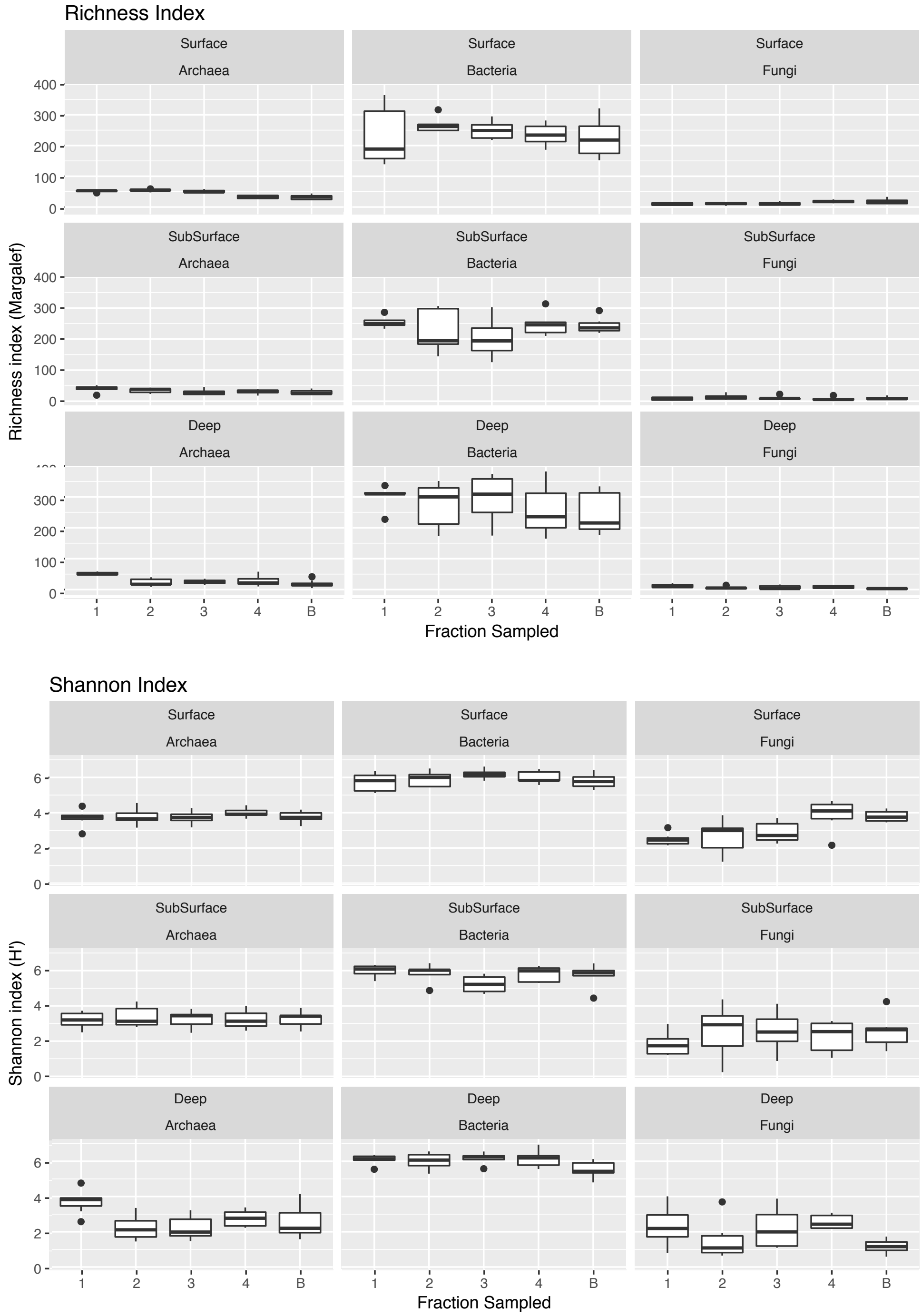

Supplementary Figure S1. Archaeal, bacterial and fungal species richness and alpha-diversity.

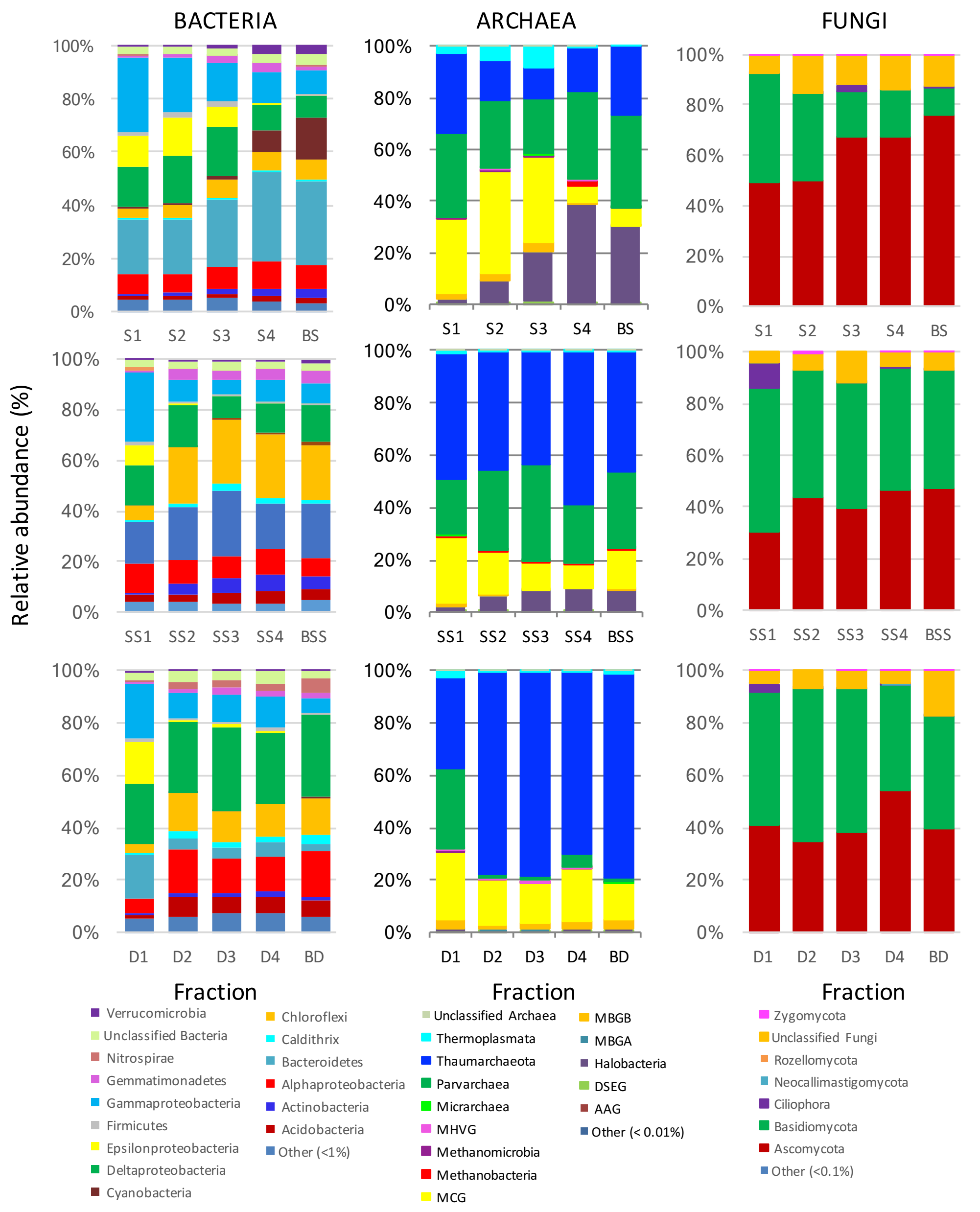

Supplementary Figure S2. Taxonomic classification of sediment bacterial and archaeal communities (OTU ≥ 97%) based on 16S rRNA gene sequencing and fungal communities based on ITS-region sequencing. For *Bacteria*, phyla classified within Other (with less than 1% contribution to total community composition) include: *Thermi*, *Ac1*, *Anck6*, *Armatimonadetes*, *Bhi180-139*, *Cd12*, *Deferribacteres*, *FCPU4*, *GAL15*, *GOUTA4*, *H-178*, *Hyd24-12*, *Kazan-3B28*, *Lcp-89*, *Ld1*, *Mvp-21*, *Mvs-104*, *Op1*, *Op9*, *Pauc34*, *Poribacteria*, *Sr1*, *Synergistetes*, *Tpd-58*, *Wps-2*, *Ws5*, *Ws6*, *Wwe1*, *Zb3* and *Betaproteobacteria*. For *Archaea*, phyla classified within Other (with less than 0.11% contribution to total community composition) include: *Archaeoglobi* and *Methanococci*. For *Fungi*, phyla classified within Other (with less than 0.11% contribution to total community composition) include: *Glomeromycota*, *Cercozoa*, *Chlorophyta* and *Chytridiomycota*.

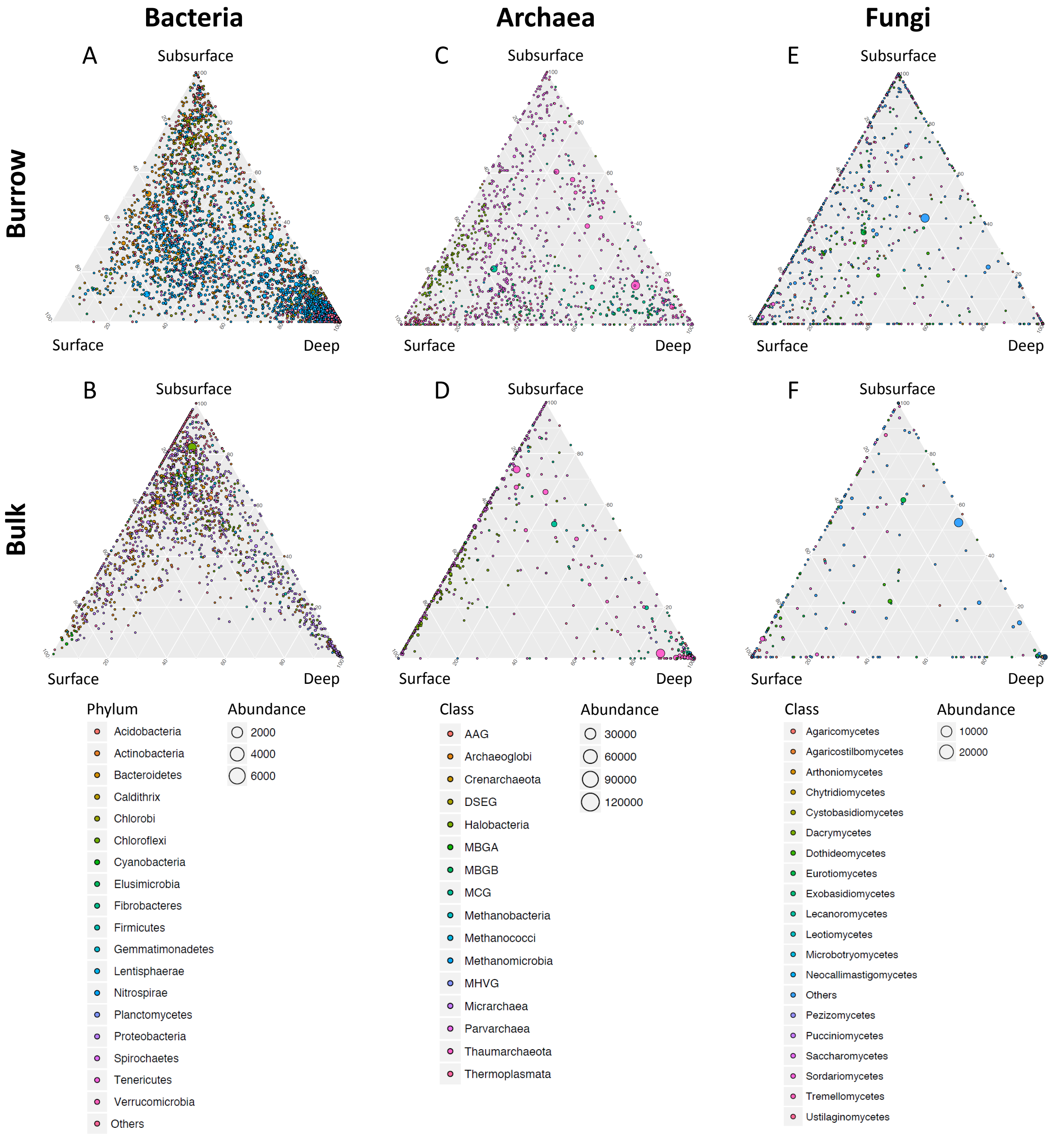

Supplementary Figure S3. Ternary plots of all OTUs detected (relative abundance > 0.01% in at least one sample) in surface, subsurface and deep sediment in the burrow and bulk for bacteria, archaea and fungi. Relative abundance of each OTU (log transformed - weighted average) is represented by each circle, with contribution of surface, subsurface and deep to total relative abundance. Circle size is proportional to mean abundance within the total sediment community. Axes indicate the contribution of each depth level. Phyla are represented by colour code.

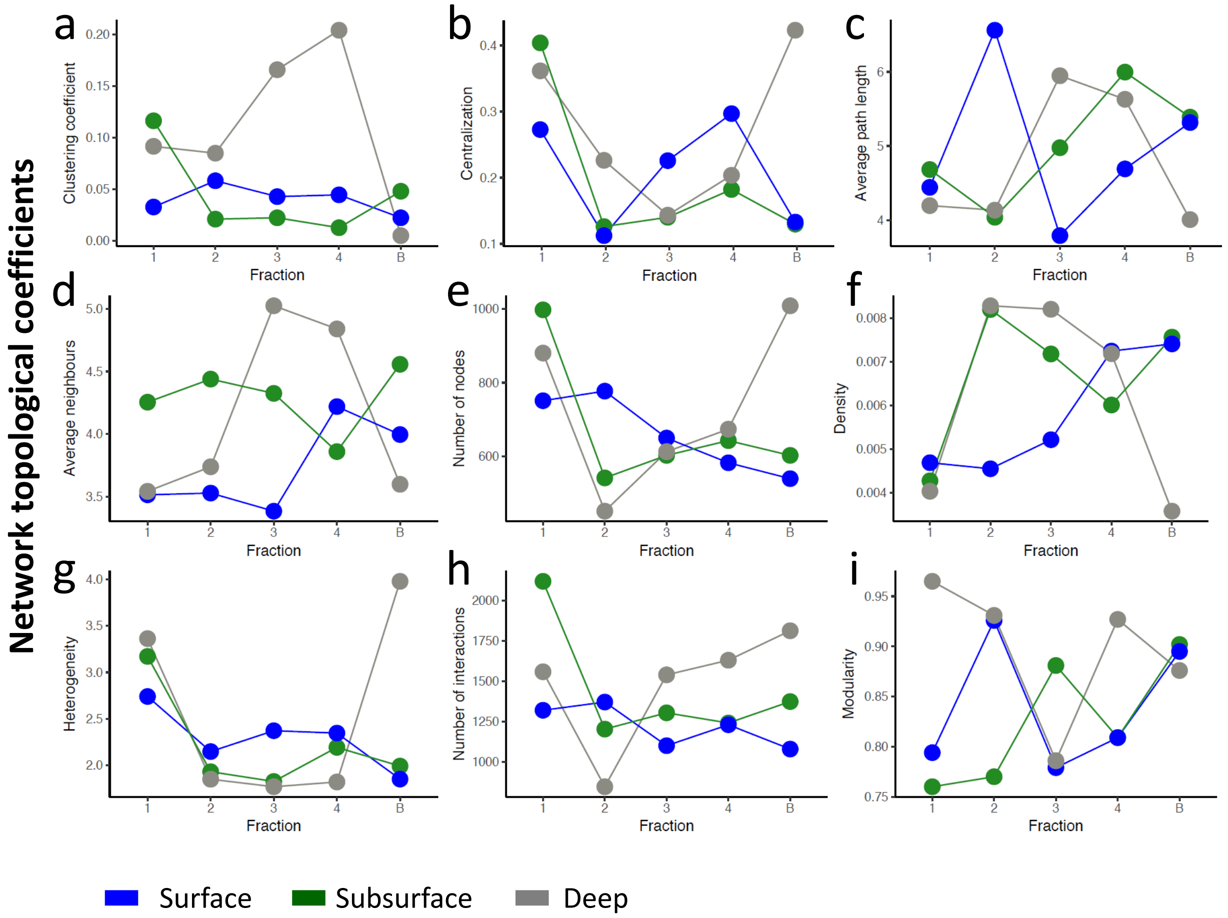

Supplementary Figure S4. Co-occurrence network analysis interaction topological coefficients for each 'Fraction'-'Depth' (a-i)

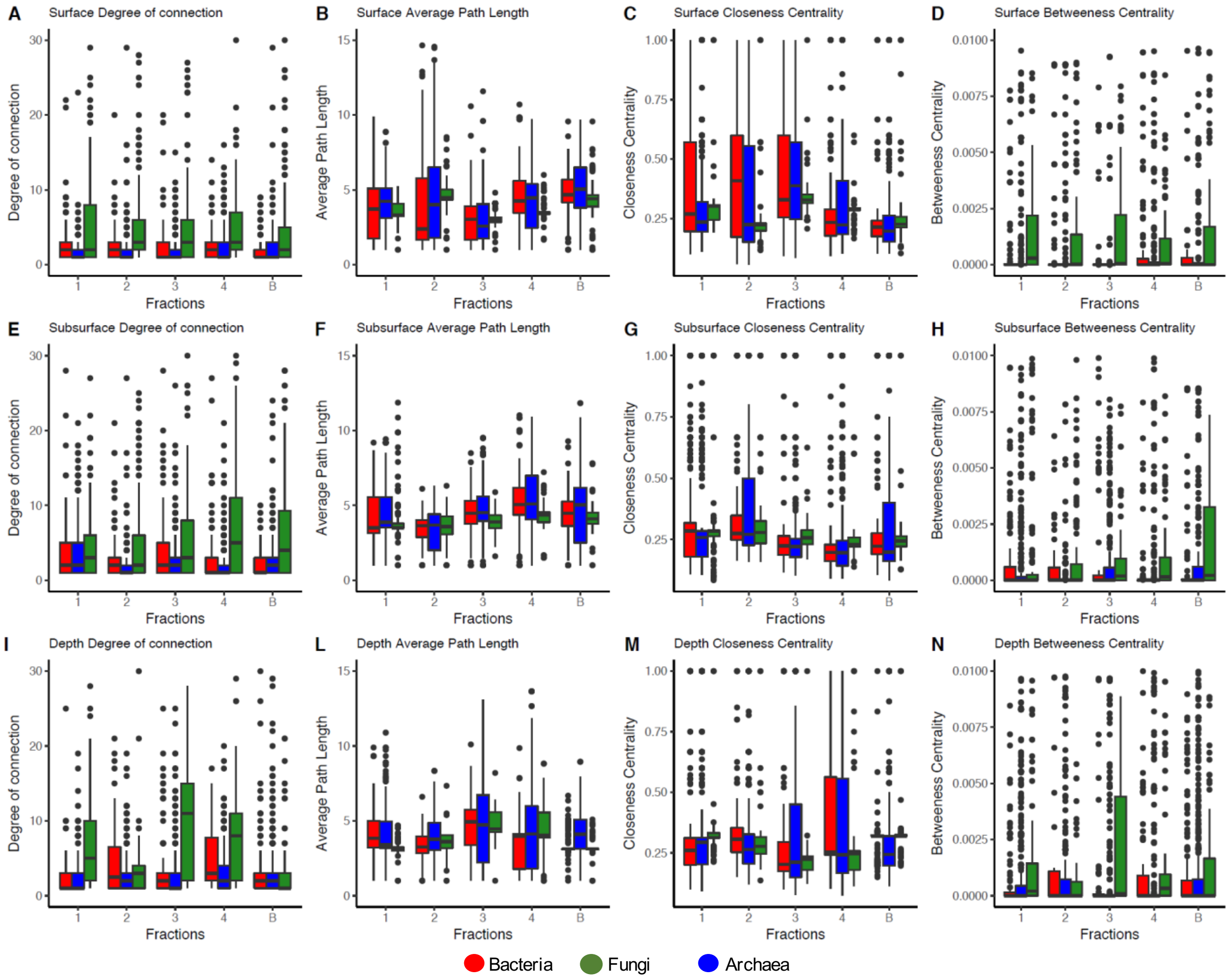

Supplementary Figure S5. Inter-Kingdom centrality measures of the microbial networks. Degree of connection, average path length, closeness centrality and the betweenness centrality are shown for each 'Depth': surface (A-D), subsurface (E-H) and deep (I-N).

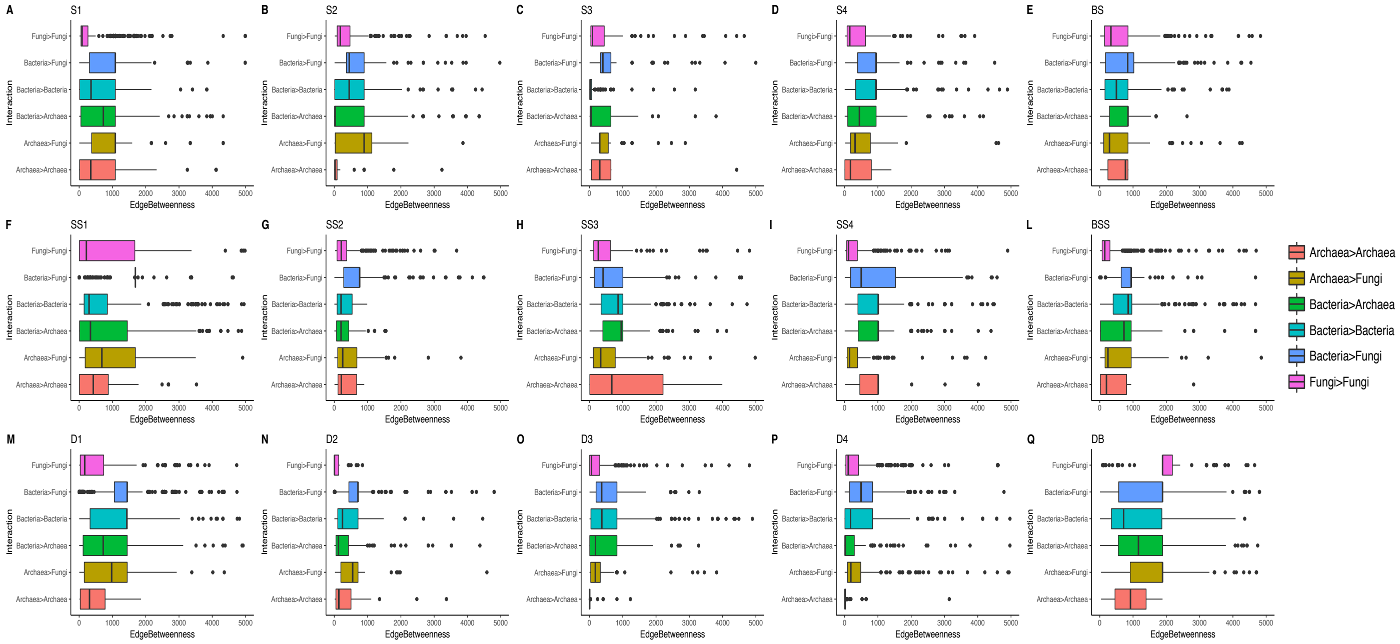

Supplementary Figure S6. Inter-Kingdom edge betweenness measures for each 'Fraction' of the surface (A-E), subsurface (F-L) and deep (M-Q).

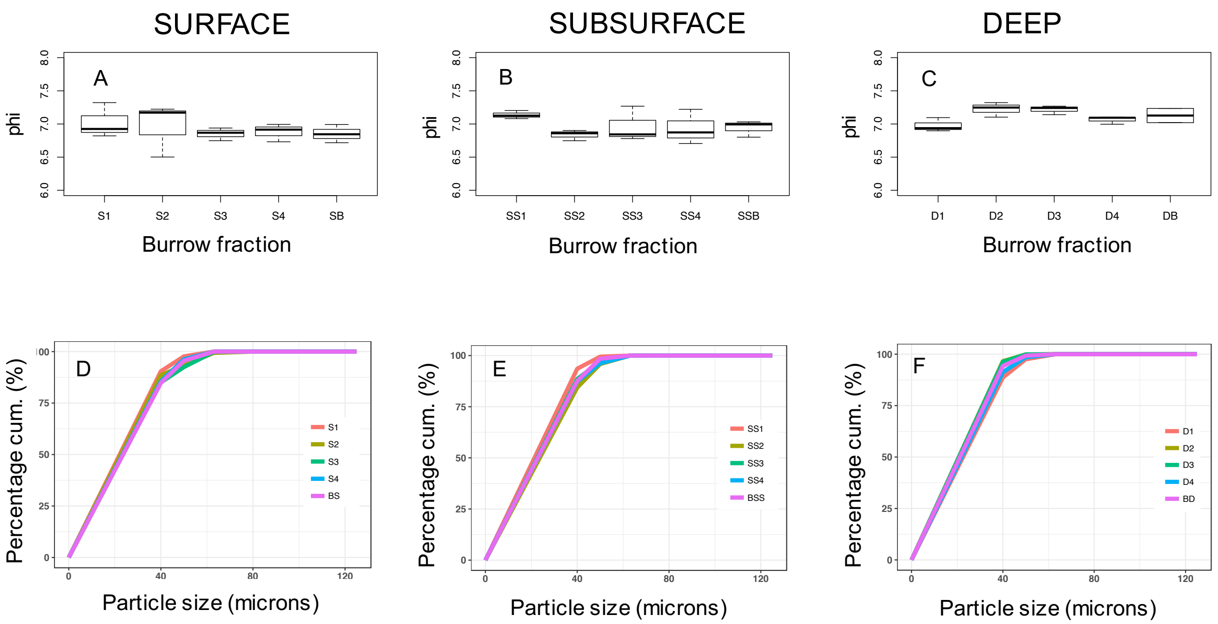

Supplementary Figure S7. Sediment Phi distribution in the surface, subsurface and deep across each 'Fraction' (A-C), with the relative distribution of the particle size (D-F).

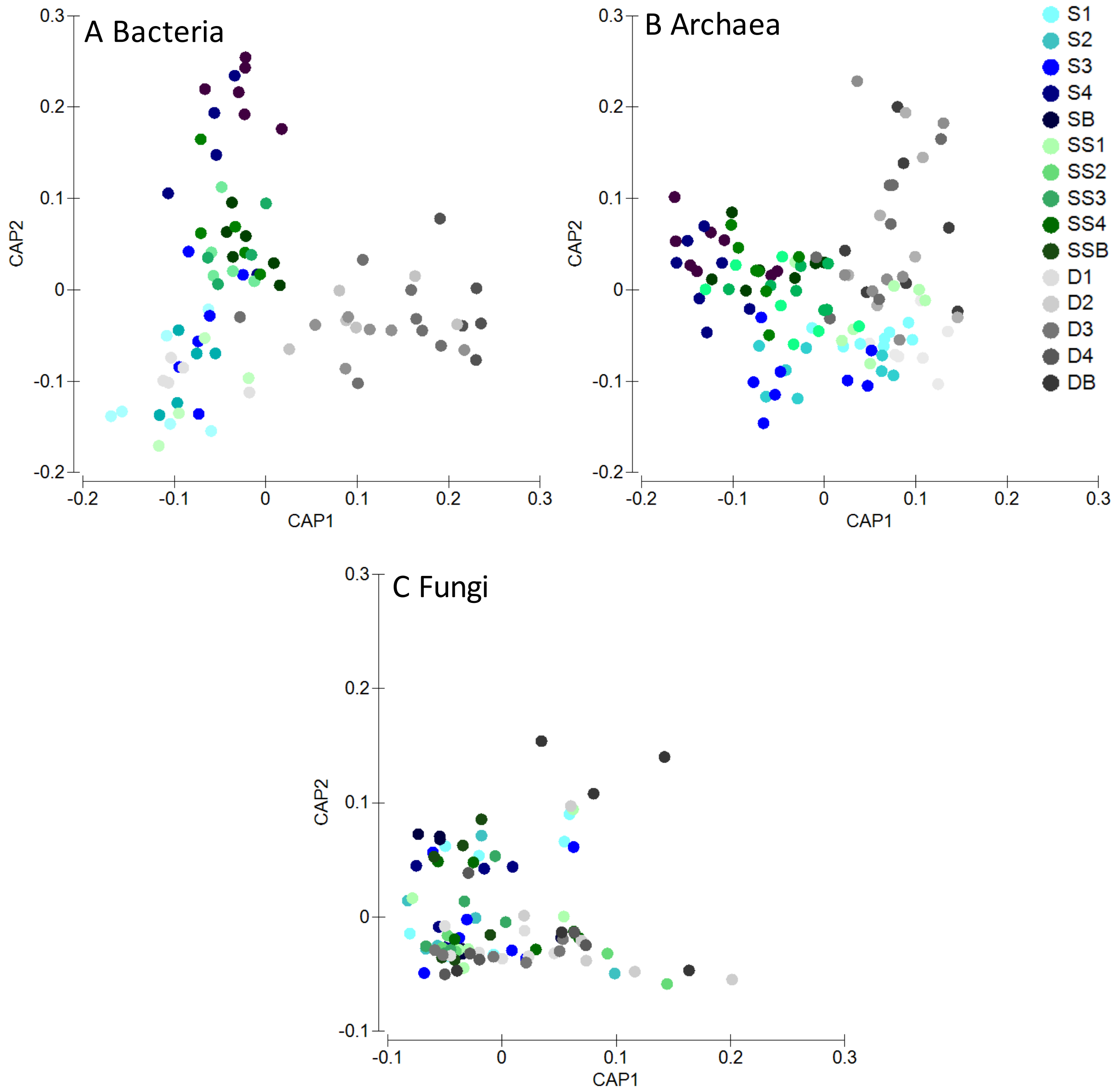

Supplementary Figure S8. Canonical analysis of principal coordinates of assignment of (a) bacterial, (b) archaeal and (c) fungal OTU function according with each 'Depth' x 'Fraction'. S, SS and D correspond to depths: surface, subsurface and deep. 1, 2, 3, 4 and B correspond to burrow fractions.

Supplementary Table S1. General linear model (GLM) testing the effect of the factors 'Depth' and 'Fraction', and their interaction, on bacterial, archaeal and fungal OTU assemblages. Res. Df = residual degrees of freedom. Df.diff = Differential degree of freedom, Dev = deviance, Pr(>Dev) = *p* statistic. Statistically significant *p* values are highlighted in bold.

BACTERIA	Res.Df	Df.diff	Dev	Pr(>Dev)
Depth	78	2	108233	**0.001**
Fraction	74	4	108428	**0.001**
Depth × Fraction	66	8	105606	**0.001**

ARCHAEA	Res.Df	Df.diff	Dev	Pr(>Dev)
Depth	106	2	15671	**0.001**
Fraction	102	4	17266	**0.001**
Depth × Fraction	94	8	20700	**0.001**

FUNGI	Res.Df	Df.diff	Dev	Pr(>Dev)
Depth	93	2	5773	**0.001**
Fraction	89	4	8709	**0.001**
Depth × Fraction	81	8	6558	**0.009**

Supplementary Table S2. Canonical Analysis of Principal components cross-validation table of bacterial, archaeal and fungal OTU assemblages for each 'Fraction' at each 'Depth'. Choice of m is the choice of the minimum number of axes based on the minimum misclassification error or minimum residual sum of squares, delta_1^2 is the first squared canonical correlation and *p* is the *p* statistic (bold indicates significance).

Kingdom	Depth	Choice of m	delta_1^2	*p*
Bacteria	Surface	7	0.9597	**0.0001**
	Subsurface	10	0.9786	**0.0002**
	Deep	9	0.8689	**0.0008**
Archaea	Surface	5	0.9578	**0.0001**
	Subsurface	11	0.9060	**0.001**
	Deep	10	0.8095	**0.001**
Fungi	Surface	5	0.6064	**0.0082**
	Subsurface	10	0.5634	0.5416
	Deep	6	0.5876	**0.0437**

Supplementary Table S3. ANOVA table for bacteria, archaea and fungi OTU diversity (A-C respectively) and richness (E-G respectively). Df= degree of freedom, Res= residual, MS = mean of square, F=F statistic and *p* is the *p* statistic. Statistically significant *p* values are highlighted in bold.

DIVERSITY

(A) BACTERIA

Source	df	MS	F	*p*
Depth	2	0.53	2.40	0.101
Fraction	4	0.21	0.94	0.441
Depth × Fraction	8	0.34	1.53	0.156
Res	66	0.22		
Total	80			

(B) ARCHAEA

Source	df	MS	F	*p*
Depth	2	12.21	47.45	**0.001**
Fraction	4	0.79	3.07	**0.012**
Depth × Fraction	8	1.08	4.18	**0.001**
Res	94	0.26		
Total	108			

(C) FUNGI

Source	df	MS	F	*p*
Depth	2	10.82	13.58	**0.001**
Fraction	4	1.44	1.81	0.117
Depth × Fraction	8	2.01	2.53	**0.014**
Res	81	0.81		
Total	95			

RICHNESS

(E) BACTERIA

Source	df	MS	F	*p*
Depth	2	11784	2.99	0.052
Fraction	4	1072	0.27	0.901
Depth × Fraction	8	2650.6	0.67	0.712
Res	66	3943.4		
Total	80			

(F) ARCHAEA

Source	df	MS	F	*p*
Depth	2	2817	42.13	**0.001**
Fraction	4	1591.6	23.80	**0.001**
Depth × Fraction	8	411.2	6.15	**0.001**
Res	94	66.87		
Total	108			

(G) FUNGI

Source	df	MS	F	*p*
Depth	2	290.6	9.04	**0.003**
Fraction	4	13.52	0.42	0.765
Depth × Fraction	8	94.80	2.95	**0.009**
Res	81	32.13		
Total	95			

Supplementary Table S4. Post-hoc pairwise tests of OTU diversity among the levels of the factor 'Fraction' at each depth for Archaea (A-C) and Fungi (E-G). T = t test, *p* = *p* statistic. Statistically significant *p* values are highlighted in bold.

ARCHAEA

(A) SURFACE

Groups	t	*p*
1, 2	0.32097	0.759
1, 3	6.89E-02	0.938
1, 4	1.6216	0.133
1, B	0.29758	0.76
2, 3	0.28187	0.784
2, 4	1.2416	0.231
2, B	8.04E-02	0.937
3, 4	1.7909	0.101
3, B	0.25376	0.821
4, B	1.7554	0.1

(B) SUBSURFACE

Groups	t	*p*
1, 2	0.68113	0.518
1, 3	0.16128	0.877
1, 4	9.34E-02	0.936
1, B	0.15666	0.879
2, 3	0.53376	0.56
2, 4	0.56964	0.554
2, B	0.55259	0.6
3, 4	6.02E-02	0.942
3, B	8.88E-03	0.993
4, B	5.32E-02	0.953

(C) DEEP

Groups	t	*p*
1, 2	3.9379	**0.002**
1, 3	3.9939	**0.001**
1, 4	3.0016	**0.013**
1, B	4.0946	**0.002**
2, 3	2.34E-02	0.986
2, 4	1.6094	0.122

FUNGI

(E) SURFACE

Groups	t	*p*
1, 2	0.37785	0.694
1, 3	1.6315	0.138
1, 4	3.6716	**0.008**
1, B	7.0042	**0.001**
2, 3	0.69252	0.517
2, 4	2.4318	**0.03**
2, B	3.0199	**0.012**
3, 4	2.2566	0.052
3, B	3.3152	**0.009**
4, B	0.12819	0.894

(F) SUBSURFACE

Groups	t	*p*
1, 2	1.0756	0.313
1, 3	1.3096	0.223
1, 4	0.89457	0.38
1, B	1.5065	0.16
2, 3	1.49E-02	0.983
2, 4	0.42451	0.67
2, B	6.32E-02	0.956
3, 4	0.4897	0.636
3, B	5.48E-02	0.952
4, B	0.51657	0.626

(G) DEEP

Groups	t	*p*
1, 2	1.4229	0.191
1, 3	0.21281	0.814
1, 4	0.47033	0.646
1, B	2.4757	**0.027**
2, 3	1.0975	0.295
2, 4	2.2272	**0.042**

2, B	0.20863	0.862	2, B	0.74374	0.463
3, 4	1.6251	0.153	3, 4	0.69466	0.52
3, B	0.18791	0.865	3, B	2.0211	0.083
4, B	1.5641	0.151	4, B	5.7814	**0.001**

Supplementary Table S5 –Post-hoc pairwise tests of OTU richness among the levels of the factor 'Fraction' at each depth for Archaea (A-C) and Fungi (E-G). T = t test, *p* = *p* statistic. Statistically significant *p* values are highlighted in bold.

ARCHAEA

(A)
SURFACE

Groups	t	*p*
1, 2	2.089	**0.056**
1, 3	1.0732	0.302
1, 4	7.5553	**0.001**
1, B	6.9684	**0.001**
2, 3	2.7375	**0.015**
2, 4	9.3016	**0.001**
2, B	8.2209	**0.001**
3, 4	5.9577	**0.001**
3, B	5.8174	**0.001**
4, B	0.56715	0.579

(B)
SUBSURFACE

Groups	t	*p*
1, 2	1.1549	0.257
1, 3	2.2633	0.05
1, 4	1.9736	0.079
1, B	2.4225	**0.024**
2, 3	1.3832	0.167
2, 4	0.95513	0.359
2, B	1.5148	0.158
3, 4	0.56527	0.567
3, B	1.42E-02	0.988
4, B	0.61291	0.55

DEEP

Groups	t	*p*
1, 2	5.8161	**0.001**
1, 3	8.0726	**0.001**
1, 4	3.6014	**0.003**
1, B	10.819	**0.001**
2, 3	0.25215	0.82
2, 4	0.54933	0.614

FUNGI

(A)
SURFACE

Groups	t	*p*
1, 2	0.29087	0.772
1, 3	0.31225	0.777
1, 4	3.1051	**0.01**
1, B	2.1037	0.066
2, 3	5.08E-02	0.954
2, 4	2.8782	**0.018**
2, B	1.935	0.075
3, 4	2.523	**0.028**
3, B	1.8033	0.094
4, B	3.73E-02	0.973

SUBSURFACE

Groups	t	*p*
1, 2	1.2229	0.274
1, 3	0.59085	0.551
1, 4	0.13879	0.893
1, B	0.52978	0.595
2, 3	0.70733	0.512
2, 4	1.294	0.212
2, B	0.94315	0.37
3, 4	0.69313	0.511
3, B	0.16326	0.876
4, B	0.65471	0.51

DEEP

Groups	t	*p*
1, 2	2.2744	**0.046**
1, 3	1.3424	0.218
1, 4	0.88329	0.417
1, B	3.3789	**0.008**
2, 3	0.53069	0.624
2, 4	1.2841	0.253

2, B	1.6558	0.13
3, 4	0.42421	0.682
3, B	2.5321	**0.026**
4, B	1.8748	0.085

2, B	1.3075	0.206
3, 4	0.52667	0.613
3, B	1.43	0.174
4, B	2.4622	**0.026**

Supplementary Table S6. Kruskall Wallis pairwise tests of the contribution of different taxa to overall community composition across the factor 'Depth' (surface, subsurface, deep).

(A) BACTERIA	χ^2	df	*p*
Unclassified *Bacteria*	32.53	2	**<0.0001**
Acidobacteria	34.53	2	**<0.0001**
Actinobacteria	22.65	2	**<0.0001**
Bacteroidetes	46.27	2	**<0.0001**
Caldithrix	27.59	2	**<0.0001**
Chloroflexi	21.95	2	**<0.0001**
Cyanobacteria	27.49	2	**<0.0001**
Firmicutes	12.29	2	**0.002**
Gemmatimonadetes	22.09	2	**<0.0001**
Nitrospirae	45.52	2	**<0.0001**
Verrucomicrobia	25.82	2	**<0.0001**
Unassigned	1.67	2	0.433
Alphaproteobacteria	8.22	2	**0.016**
Deltaproteobacteria	35.72	2	**<0.0001**
Epsilonproteobacteria	10.61	2	**0.005**
Gammaproteobacteria	12.06	2	**0.002**

(B) ARCHAEA	χ^2	df	*p*
Unclassified *Archaea*	8.03	2	**0.018**
Micrarchaea	4.17	2	0.125
Parvarchaea	40.70	2	**<0.0001**
AAG	9.19	2	**0.01**
Other	3.26	2	0.196
DSEG	19.87	2	**<0.0001**
Halobacteria	61.13	2	**<0.0001**
MBGA	36.65	2	**<0.0001**
MBGB	7.65	2	**0.022**
MCG	3.83	2	0.147
Methanobacteria	12.64	2	**0.002**
Methanomicrobia	9.28	2	**0.011**
MHVG	23.22	2	**<0.0001**
Thaumarchaeota	41.03	2	**<0.0001**
Thermoplasmata	7.55	2	**0.023**

(C) FUNGI	χ^2	df	*p*
Unclassified *Fungi*	2.36	2	0.307
Ascomycota	12.90	2	**0.002**
Basidiomycota	13.91	2	**0.001**
Ciliophora	1.29	2	0.524
Neocallimastigomycota	0.42	2	0.810
Rozellomycota	2.66	2	0.265
Zygomycota	3.58	2	0.167

Supplementary Table S7. Kruskall Wallis pairwise tests of the contribution of different (A) bacterial, (B) archaeal and (C) fungal taxa to overall community composition across the factor 'Fraction' within each 'Depth' (surface, subsurface, deep).

	Surface			Subsurface			Deep		
(A) BACTERIA	χ^2	df	*p*	χ^2	df	*p*	χ^2	df	*p*
Unclassified *Bacteria*	6.20	4	0.185	4.60	4	0.331	8.42	4	0.077
Acidobacteria	4.13	4	0.389	3.34	4	0.502	10.59	4	**0.032**
Actinobacteria	14.79	4	**0.005**	10.10	4	**0.039**	4.07	4	0.396
Bacteroidetes	8.14	4	0.087	3.34	4	0.502	10.54	4	**0.032**
Caldithrix	1.62	4	0.804	1.44	4	0.837	13.35	4	**0.010**
Chloroflexi	6.30	4	0.178	6.31	4	0.177	11.33	4	**0.023**
Cyanobacteria	17.78	4	**0.001**	7.01	4	0.135	6.30	4	0.178
Firmicutes	17.82	4	**0.001**	7.50	4	0.112	6.92	4	0.140
Gemmatimonadetes	12.53	4	**0.014**	9.97	4	**0.041**	8.63	4	0.071
Nitrospirae	12.95	4	**0.012**	9.08	4	0.059	12.66	4	**0.013**
Verrucomicrobia	20.59	4	**0.0004**	7.65	4	0.105	7.01	4	0.136
Unassigned	9.23	4	0.056	4.44	4	0.350	5.87	4	0.209
Alphaproteobacteria	10.47	4	**0.033**	4.44	4	0.350	10.46	4	**0.033**
Deltaproteobacteria	13.69	4	**0.008**	5.14	4	0.273	3.29	4	0.511
Epsilonproteobacteria	20.98	4	**0.0003**	14.75	4	**0.005**	15.22	4	**0.004**
Gammaproteobacteria	22.30	4	**0.0002**	11.24	4	**0.024**	14.09	4	**0.007**

	Surface			Subsurface			Deep		
(B) ARCHAEA	χ^2	df	*p*	χ^2	df	*p*	χ^2	df	*p*
Unclassified *Archaea*	18.54	4	**0.001**	5.08	4	0.279	5.32	4	0.256
Microarchaea	16.07	4	**0.003**	8.58	4	0.072	6.49	4	0.166
Parvarchaea	6.14	4	0.189	3.22	4	0.521	16.11	4	**0.003**
AAG	24.11	4	**0.00008**	3.90	4	0.420	7.08	4	0.132
Other	16.02	4	**0.003**	2.95	4	0.566	5.23	4	0.265
DSEG	26.65	4	**0.00002**	15.80	4	**0.003**	8.39	4	0.078
Halobacteria	22.97	4	**0.0001**	14.48	4	**0.006**	9.37	4	0.053
MBGA	19.86	4	**0.001**	8.32	4	0.080	1.27	4	0.866
MBGB	26.61	4	**0.00002**	11.17	4	**0.025**	5.60	4	0.231
MCG	26.17	4	**0.00003**	5.87	4	0.209	4.33	4	0.363
Methanobacteria	7.45	4	0.114	5.76	4	0.218	3.22	4	0.521
Methanomicrobia	29.50	4	**0.00001**	13.27	4	**0.010**	17.85	4	**0.001**
MHVG	24.18	4	**0.00007**	9.91	4	**0.042**	2.22	4	0.695
Thaumarchaeota	11.14	4	**0.025**	1.56	4	0.817	8.56	4	0.073
Thermoplasmata	26.72	4	**0.00002**	20.10	4	**0.0005**	12.01	4	**0.017**

	Surface			Subsurface			Deep		
(C) FUNGI	χ^2	df	*p*	χ^2	df	*p*	χ^2	df	*p*
Unclassified *Fungi*	7.10	4	0.131	1.07	4	0.899	0.80	4	0.938
Ascomycota	8.26	4	0.083	2.39	4	0.664	3.53	4	0.473
Basidiomycota	8.57	4	0.073	0.45	4	0.979	2.10	4	0.718
Ciliophora	3.73	4	0.444	2.43	4	0.658	4.09	4	0.395
Neocallimastigomycota	2.02	4	0.733	5.77	4	0.217	4.61	4	0.330
Rozellomycota	5.75	4	0.218	3.35	4	0.502	4.46	4	0.348
Zygomycota	2.87	4	0.580	4.20	4	0.380	3.38	4	0.496

Supplementary Table S8. Two-way PERMANOVA to test effect of 'Depth' and 'Fraction on **(A)** Biogeochemistry, **(B)** metal content and **(C)** grain size. 'Depth' (3 levels: surface, subsurface, deep); 'Burrow' (2 levels: bulk, burrow). Significant effects and interactions are highlighted in bold. Df: degrees of freedom; MS: mean sum of squares; F: F statistic value; *P*: *P* statistic for Monte-Carlo test. Statistically significant *p* values are highlighted in bold.

(A) Biochemistry

Source	df	MS	F	*P*
Depth	2	28.822	4.0792	**0.001**
Fraction	4	17.252	2.4418	**0.006**
Depth x Fraction	8	7.1725	1.0151	0.456
Res	30	7.0656		
Total	44			

(B) Metals

Source	df	MS	F	*P*
Depth	2	13.42	1.8014	0.099
Fraction	4	3.0191	0.40524	0.966
Depth x Fraction	8	5.6978	0.7648	0.769
Res	30	7.45		
Total	44			

(C) Grain size

Source	df	MS	F	*P*
Depth	2	37.109	3.7824	**0.002**
Fraction	4	10.224	1.0421	0.418
Depth x Fraction	8	13.168	1.3422	0.136
Res	30	9.811		
Total	44			

Supplementary Table S9. SIMPER analysis of the contribution of geochemical variables to the environmental dissimilarity among different sediment depths: (A) Surface and Subsurface, (B) Surface and Deep and (C) Subsurface and Deep. Contrib% = % contribution of variable to dissimilarity among samples. Cum. % = % cumulative contribution of variable to dissimilarity amongst samples. PIC = particulate inorganic carbon; POC = particulate organic carbon; PIN = particulate inorganic nitrogen.

(A) Surface and Subsurface			(B) Surface and Deep			(C) Subsurface and Deep		
Variable	Contrib%	Cum.%	Variable	Contrib%	Cum.%	Variable	Contrib%	Cum.%
PIC	28.16	28.16	PIC	25.71	25.71	Silicate	31.26	31.26
Sulphate	27.46	55.61	Sulphate	21.36	47.06	POC	22.98	54.24
POC	17.35	72.96	POC	16.77	63.83	Sulphate	22.64	76.88
PIN	15.03	88	Silicate	13.54	77.37	Phosphate	15.54	92.42
Silicate	7.54	95.54	PIN	13.32	90.69			

Supplementary Table S10. SIMPER analysis of the contribution of geochemical variables to the environmental dissimilarity among different burrow fractions Contrib% = % contribution of variable to dissimilarity amongst samples. Cum. % = % cumulative contribution of variable to dissimilarity amongst samples. PIC = particulate inorganic carbon; POC = particulate organic carbon; PIN = particulate inorganic nitrogen.

Fractions 1 and 2			Fractions 1 and 3			Fractions 1 and 4		
Variable	Contrib%	Cum.%	Variable	Contrib%	Cum.%	Variable	Contrib%	Cum.%
PIC	35.89	35.89	PIC	35.55	35.55	PIC	32.59	32.59
POC	19.16	55.05	POC	19.93	55.48	POC	23.29	55.88
Sulphate	15.83	70.88	Sulphate	17.81	73.28	PIN	16.36	72.24
PIN	12.02	82.9	PIN	10.2	83.48	Silicate	11.02	83.26
Phosphate	9.11	92.02	Phosphate	7.94	91.41	Phosphate	8.72	91.97

Fractions 1 and Bulk			Fractions 2 and 3			Fractions 2 and 4		
Variable	Contrib%	Cum.%	Variable	Contrib%	Cum.%	Variable	Contrib%	Cum.%
PIC	28.63	28.63	Sulphate	44.24	44.24	Sulphate	29.17	29.17
POC	28.38	57.01	Silicate	19.52	63.75	Silicate	24.06	53.23
Sulphate	15.57	72.57	POC	17.41	81.17	PIN	17.16	70.39
PIN	11.12	83.69	Phosphate	11.11	92.27	POC	12.2	82.59
Silicate	8.19	91.88				Phosphate	10.38	92.97

Fractions 2 and Bulk			Fractions 3 and 4			Fractions 3 and Bulk		
Variable	Contrib%	Cum.%	Variable	Contrib%	Cum.%	Variable	Contrib%	Cum.%
Sulphate	45.21	45.21	Sulphate	29.94	29.94	Sulphate	39.86	39.86
POC	21.53	66.74	Silicate	25.86	55.8	Silicate	25.18	65.04
Silicate	19.13	85.87	PIN	17.48	73.28	POC	23.55	88.59
Phosphate	10	95.87	POC	13.46	86.74	PIN	3.77	92.36
			PIC	7.92	94.65			

Fractions 4 and Bulk		
Variable	Contrib%	Cum.%
Silicate	33.62	33.62
Sulphate	32.69	66.31
PIN	18.98	85.28
PIC	5.69	90.98

Supplementary Table S11. ANOVA results table showing significant effects of 'Depth' and 'Fraction' on geochemical variables.

Variable	Source	F:df1,df2	P
PIC	Depth	3.81:2,44	0.0379
Nitrite	Depth	9.2:2,44	0.0008
Sulphate	Depth	10.1:2,44	0.0002
	Fraction	3.03:4,44	0.0319
POC	Fraction	7.4:4,44	0.0005
Nitrate	Fraction	1.99:4,44	0.09
Silicate	Fraction	1.13:4,44	0.0307

Supplementary Table S12 - SIMPER analysis of grain size classes contributing dissimilarity amongst sediment depths. Contrib% = % contribution of variable to dissimilarity amongst samples. Cum. % = % cumulative contribution of variable to dissimilarity amongst samples. S and SS (surface and subsurface), S and D (surface and deep), SS and D (subsurface and deep).

S and SS	Contrib.%	Cum.%	S and D	Contrib.%	Cum.%	SS and D	Contrib.%	Cum.%
48.6	7	7	50.8	7.94	7.94	46.4	9.94	9.94
50.8	6.92	13.93	48.6	7.71	15.65	48.6	9.03	18.97
46.4	6.55	20.48	53.2	6.93	22.57	44.4	8.97	27.94
53.2	6.28	26.76	46.4	6.44	29.01	42.4	6.84	34.78
44.4	5.68	32.44	55.6	5.29	34.3	50.8	6.52	41.31
55.6	5.19	37.63	44.4	4.89	39.19	40.6	4.68	45.99
42.4	4.65	42.28	42.4	3.85	43.05	53.2	3.64	49.62
40.6	3.86	46.14	58.2	3.61	46.65	31.0	3.43	53.05
58.2	3.8	49.94	40.6	3.52	50.17	32.4	3.42	56.48
38.8	3.38	53.32	38.8	3.51	53.68	38.8	3.2	59.68
45.0	3.03	56.36	37.0	3.36	57.04	33.8	3	62.68
44.5	2.7	59.05	31.0	3.08	60.12	29.6	2.85	65.53
44.0	2.66	61.71	29.6	3.04	63.16	35.4	2.57	68.1
43.4	2.65	64.37	35.4	2.99	66.16	37.0	2.56	70.66
42.9	2.43	66.8	32.4	2.83	68.99	28.2	1.97	72.63
42.4	2.39	69.18	33.8	2.75	71.74	55.6	1.51	74.14
60.8	2.34	71.52	28.2	2.53	74.27	27.0	1.28	75.42
28.2	2.28	73.8	60.8	2.12	76.39	23.6	1.23	76.65
27.0	1.55	75.36	27.0	1.75	78.14	24.6	1.14	77.8
63.6	1.12	76.47	25.8	1.11	79.25	22.6	1.11	78.91
21.6	1.09	77.56	63.6	0.99	80.24	25.8	1.06	79.96
22.6	1.05	78.61	22.6	0.94	81.18	12.0	0.93	80.89
20.6	0.98	79.59	23.6	0.9	82.08	11.4	0.91	81.8
25.8	0.94	80.53	24.6	0.86	82.94	21.6	0.87	82.67
23.6	0.85	81.38	21.6	0.83	83.76	17.2	0.83	83.5
11.4	0.85	82.23	20.6	0.6	84.36	12.5	0.82	84.32
12.0	0.83	83.06	10.9	0.57	84.93	16.4	0.8	85.12
19.7	0.82	83.88	11.4	0.56	85.49	18.0	0.78	85.9
10.9	0.77	84.65	10.5	0.53	86.02	10.9	0.75	86.65
12.5	0.74	85.39	12.0	0.5	86.52	15.7	0.7	87.36
18.8	0.73	86.12	15.7	0.5	87.02	18.8	0.67	88.03
24.6	0.72	86.85	16.4	0.47	87.5	20.6	0.67	88.7
18.0	0.7	87.55	15.0	0.47	87.96	13.1	0.65	89.35

17.2	0.7	88.25	10.0	0.46	88.42	19.7	0.61	89.96
16.4	0.69	88.94	12.5	0.42	88.84	15.0	0.59	90.55
15.7	0.68	89.61	17.2	0.4	89.24			
13.1	0.66	90.27	14.3	0.4	89.64			
			19.7	0.39	90.03			

Supplementary Table S13. DistLM showing the main drivers of bacterial and archaeal beta-diversity in terms of (A) biogeochemistry and (B) grain size (note: no significant drivers were detected for *Fungi*). Explanatory variables are shown in the first column. AICc: Akaike Information Criterion value; SS: Sum of square; Pseudo-F: F statistic; *P*: *P* statistic; Prop.: variance explained by the single variable; Cumul.: cumulative variance explained; res.df: residual degree of freedom.

(A) Biochemistry

Bacteria	AICc	SS(trace)	Pseudo-F	*P*	Prop.	Cumul.	res.df
POC (+)	343.88	9221.9	4.6522	0.0001	9.76E-02	9.76E-02	43
Nitrite (+)	342.96	5882.6	3.1135	0.0006	6.23E-02	0.15991	42
Nitrate (+)	342.46	4975.9	2.7429	0.0013	5.27E-02	0.21258	41
**Archaea**	**AICc**	**SS(trace)**	**Pseudo-F**	***P***	**Prop.**	**Cumul.**	**res.df**
Nitrate (+)	328.16	8075.9	5.7771	0.0001	0.11844	0.11844	43
POC (+)	325.42	6367.3	4.976	0.0001	9.34E-02	0.21182	42
PON (+)	325.04	3243	2.6329	0.0061	4.76E-02	0.25938	41

(B) Grain size

Archaea	AICc	SS(trace)	Pseudo-F	*P*	Prop.	Cumul.	res.df
1.882	329.65	6052.6	4.1887	0.0004	8.88E-02	8.88E-02	43
60.8	328.48	4612.6	3.3679	0.0013	6.76E-02	0.15641	42
0.834	328.09	3482.7	2.6424	0.0079	5.11E-02	0.20749	41
4.86	326.14	5127.1	4.1929	0.0002	7.52E-02	0.28268	40

Supplementary Table S14. 2-way PERMANOVA to test effect of 'Depth' and 'Fraction on functional group assignment for **(A)** Bacteria, **(B)** Archaea and **(C)** Fungi. 'Depth' (3 levels: surface, subsurface, deep); 'Burrow' (2 levels: bulk, burrow). Significant effects and interactions are highlighted in bold. Df: degrees of freedom; MS: mean sum of squares; F: F statistic; *P*: *P* statistic. Statistically significant *P* values are highlighted in bold.

(A) Bacteria

Source	df	MS	F	*P*
Depth	2	14964	14.92	**0.0001**
Fraction	4	3508.9	3.51	**0.0001**
Depth x Fraction	8	2064.7	2.06	**0.001**
Res	66	1003.3		
Total	80			

(B) Archaea

Source	df	MS	F	*P*
Depth	2	2520.1	30.36	**0.0001**
Fraction	4	468.53	5.65	**0.0001**
Depth x Fraction	8	202.33	2.44	**0.001**
Res	94	83		
Total	108			

(C) Fungi

Source	df	MS	F	*P*
Depth	2	1667.7	3.59	**0.0015**
Fraction	4	695.94	1.51	0.1103
Depth x Fraction	8	445.05	0.96	0.5464
Res	81	464.79		
Total	95			
